# Nanotheranostics for Image-Guided Cancer Treatment

**DOI:** 10.3390/pharmaceutics14050917

**Published:** 2022-04-22

**Authors:** Isabel S. Dennahy, Zheng Han, William M. MacCuaig, Hunter M. Chalfant, Anna Condacse, Jordan M. Hagood, Juan C. Claros-Sorto, Wajeeha Razaq, Jennifer Holter-Chakrabarty, Ronald Squires, Barish H. Edil, Ajay Jain, Lacey R. McNally

**Affiliations:** 1 Department of Surgery, University of Oklahoma Health Science Center, Oklahoma City, OK 73104, USA; isabel-dennahy@ouhsc.edu (I.S.D.); zheng-han@ouhsc.edu (Z.H.); hunter-chalfant@ouhsc.edu (H.M.C.); anna-condacse@ouhsc.edu (A.C.); jordan-hagood@ouhsc.edu (J.M.H.); juan-clarossorto@ouhsc.edu (J.C.C.-S.); ronald-squires@ouhsc.edu (R.S.); barish-edil@ouhsc.edu (B.H.E.); ajay-jain@ouhsc.edu (A.J.); 2 Department of Bioengineering, University of Oklahoma, Norman, OK 73019, USA; bmaccuaig9@gmail.com; 3 Department of Internal Medicine, University of Oklahoma Health Science Center, Oklahoma City, OK 73104, USA; wajeeha-razaq@ouhsc.edu (W.R.); jennifer-holter@ouhsc.edu (J.H.-C.)

**Keywords:** targeted drug delivery, antibody–drug conjugates, nanotheranostics, image-guided therapy, nanoparticles, drug carriers, radiolabelling, anticancer therapy

## Abstract

Image-guided nanotheranostics have the potential to represent a new paradigm in the treatment of cancer. Recent developments in modern imaging and nanoparticle design offer an answer to many of the issues associated with conventional chemotherapy, including their indiscriminate side effects and susceptibility to drug resistance. Imaging is one of the tools best poised to enable tailoring of cancer therapies. The field of image-guided nanotheranostics has the potential to harness the precision of modern imaging techniques and use this to direct, dictate, and follow site-specific drug delivery, all of which can be used to further tailor cancer therapies on both the individual and population level. The use of image-guided drug delivery has exploded in preclinical and clinical trials although the clinical translation is incipient. This review will focus on traditional mechanisms of targeted drug delivery in cancer, including the use of molecular targeting, as well as the foundations of designing nanotheranostics, with a focus on current clinical applications of nanotheranostics in cancer. A variety of specially engineered and targeted drug carriers, along with strategies of labeling nanoparticles to endow detectability in different imaging modalities will be reviewed. It will also introduce newer concepts of image-guided drug delivery, which may circumvent many of the issues seen with other techniques. Finally, we will review the current barriers to clinical translation of image-guided nanotheranostics and how these may be overcome.

## 1. Introduction

Though the field of medicine has seen unprecedented growth in the last few decades, one area in which we still face many challenges is the targeted treatment of cancers. The concept of “targeted drug delivery” in cancer promises to focus the effects of anticancer agents onto the cancer cells themselves, avoiding, as much as possible, cytotoxic effects on healthy, non-cancerous cells. This would not only aim to minimize systemic side effects but could also enable the delivery of higher drug doses locally and help to act against mechanisms of drug resistance [1,2].

One of the first ways in which targeting has been achieved in recent years is through the attachment of antibodies or antibody fragments to drugs to act as cell-recognition molecules. Antibody–drug conjugates (ADCs) bind to specific antigens known to be overexpressed in the cancer in question, and therefore actively target these cells. To date, several ADCs have been approved for use in cancers such as acute myeloid leukemia and acute lymphoblastic leukemia, various forms of lymphoma, lung cancer, gastric cancer, and breast cancer [3]. Despite the preliminary success of ADCs, however, they are often imperfect in their targeting mechanism, resulting in unique, albeit different, side effect profiles. They have also seen issues with ensuring that the drug decouples from the antibody at the right time to provide appropriate localization [4]. Moreover, they have also been slow to apply in many solid tumors due to problems with drug penetration [5]. In addition, it has been shown that high expression of HER2 by hepatocytes has also shown to facilitate ADC accumulation in the liver, resulting in hepatotoxicity [6].

Nanoparticles (NPs) are promising to improve tumor target specificity while they can be rapidly eliminated from the body. NPs offer protection to encapsulated drugs [7,8], and improve the pharmacokinetics and prolong circulation time of NP-formulated medications, without compromising the desired effect on molecular targets. Compared to ADCs that only carry 1–4 drug molecules per body [4,9], NPs have a much higher loading capacity. For example, a 2 nm gold NP can load ~100 molecules on the surface [10], and loading capacity scales with NP size. In the context of cancer, several nano-drug delivery systems (nanoDDS) of doxorubicin (Doxil, Caelyx, and Myocet), irinotecan (Onivyde), paclitaxel (Abraxane), and vincristine (Marqibo) have been clinically approved, with many others in clinical trials [11]. However, current clinically approved nanoDDS are mostly passively targeted through the enhanced permeation and retention (EPR) effect. NanoDDS reliant on the EPR effect have been shown to have an improved safety profile, and modest, if any, improvement in therapeutic efficacy [12]. The fact that an astonishingly small fraction (often < 5%) can reach the tumor sites [13,14], let alone diffuse through the vasculature and into cancer cells [15,16], necessitates the development of “actively-targeted” nanoDDS so that their capabilities to localize and retain in cancer can be enhanced. Aligned with the mission of precision medicine, nanoDDS detectable in imaging modalities, also known as nanotheranostics, have inherent advantages to answer questions about their localization.

For targeted nanoDDS, imaging serves as a “pilot” evaluation of where a targeted NPs localizes, shedding light on “on-target efficiency”. The image-guided treatment regime can also facilitate identifying patients who lack the common target and will not respond to treatment, which is critical for treatment planning. Designing nanotheranostic particles with high efficiency and translational potential demands careful choice of the composition of NPs, imaging labels to be added to the NPs, in addition to their target of choice and cargo to be delivered. In this review, we will review common NP types to construct nanotheranostics, choice of cancer targets and targeting moieties, and new strategies of NP-labelling to confer imaging detectability in different imaging modalities. We will review preclinical and clinical applications of nanotheranostics in facilitating image-guided therapies in cancer, with an emphasis on prominent examples of these nanotheranostics in clinics. 

## 2. Strategies of Constructing Nanotheranostics

**Figure 1:**
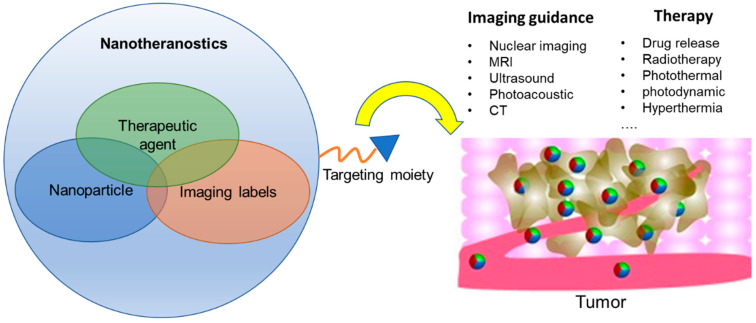
**Overview of the multifaceted imaging and therapeutic capabilities of nanotheranostics.** While nanotheranostics are composed of nanoparticle (NP), imaging labels, and therapeutic agents, the three components may overlap and NPs alone may function as imaging and therapeutic agents. Upon attaching a targeting moiety to enable active targeting to tumor, they, at the tumor site, could generate imaging contrast in a variety of imaging modalities, meanwhile release drug in a controlled manner, facilitate radiotherapy, photothermal, photodynamic, hyperthermia, etc. Therefore, nanotheranostics are extremely versatile in guiding its targeted therapy prior to efficient treatment of cancer.

Nanotheranostics offers the potential not only to facilitate the targeted delivery of drugs to cancer cells but also to utilize imaging to reveal the efficiency of drug delivery, off-target effects, potential toxicity, and further suitability of such nanotheranostics in a particular patient. Some NPs carrying imaging labels could also offer new modes of cancer treatment as they are inherently tumor antagonists, e.g., radioisotopes, or become cytotoxic under certain external localized stimuli, such as alternating magnetic gradient, light, or ultrasound, which adds to the benefit of nanotheranostics in cancer [17,18] (Figure 1). While nanotheranostics can take a variety of forms, three important design parameters of nanotheranostics are (1) nanoparticle composition, size, and shape, (2) targeting moieties and (3) imaging labels.

### 2.1. Nanoparticle Composition, Size and Shape

NPs used to construct nanotheranostics majorly involve two categories: organic NPs, and inorganic NPs. An overview of different NP types in each category, their drug payload, and clinical or preclinical stages is shown Table 1. Organic NPs include liposomes, dendrimers, and polymers. Organic NPs are the more commonly used group as they are considered biocompatible, biodegradable, easy to manufacture, and cost-effective [19]. Inorganic NPs are comprised of biologically inert inorganic materials, such as silicon, gold, silver, carbon, and iron. Inorganic NPs can form peculiar structures, e.g., porous, core–shell, rods, to facilitate loading drugs and imaging labels. Notably, some inorganic NPs are inherently imageable due to their optical and magnetic properties. For example, fluorescent gold NPs can be detected optically or in photoacoustic imaging (PAI) [20], and iron oxide NPs can be detected in MRI [21], in addition to their capacity to load drugs. The following section will introduce characteristics of NPs in each category and their potential modification strategies.

#### 2.1.1. Liposomes

Liposomes, which are nanocarriers comprised from one or more layers of natural or synthetic lipids, are commonly used nanoDDS. Each layer of lipid comprises a collection of phospholipids with hydrophilic heads and hydrophobic tails, which self-assemble in aqueous solutions [38]. Liposomes have the ability to carry either hydrophilic or hydrophobic compounds depending on the lipid bilayer construction, where hydrophilic drugs can be carried in the center of the liposome, while hydrophobic drugs can be carried between the phospholipid layers of the liposome [39,40,41]. Drugs and imaging labels can be added on the surface, within the lipid layer, or intraluminally [42,43]. For example, a PEGylated liposome with base phospholipids of lecithin and cholesterol was loaded with hydrophilic gene probe for imaging hypoxia, and also a hydrophobic photosensitizer. The liposomal delivery of probe was detected via fluorescence imaging prior to therapeutic treatment via photodynamic therapy by utilizing the delivered photosensitizer [44,45]. Further, a radiolabeled liposome formulation containing chemodrugs paclitaxel and vinorelbine has resulted in theranostic detection and treatment in a preclinical model. The inherent ability of liposomes to self-assemble, their controllable size allowing for large payloads, and versatility of surface modification. Large payloads delivered to the tumor as a result of the EPR effect leads to improved drug efficacy while minimizing off-target delivery and damage [46]. Further, surface modifications allow for active-targeting of tumor features, such as folate receptor [47] or syndecan-1 [48], for improvement over the EPR effect. Coating of liposomes with other materials, such as polymers (see below), have been investigated to further increase specificity to cancer. Such advantages have resulted in liposome formulations that are more readily applied for approval use in clinics [22].

#### 2.1.2. Polymeric Nanoparticles

Polymers can be natural or synthetic and are composed of repeating monomers [23]. Polymers are good options to form NPs because of their facile synthetic process [49] and cost-effectiveness. Their characteristics of biocompatibility, biodegradability and stability against degradation make them amenable NPs for clinical translation [50]. In general, polymers offer increased stability and cargo-loading efficiency when compared to liposomes [51,52]. Poly(lactic-co-glycolic acid) (PLGA) and polylactic acid (PLA) are examples of FDA-approved polymers with biocompatible and biodegradable properties. Intravenously administered NPs are largely cleared from the bloodstream by the mononuclear phagocyte system, including macrophages, which threatens their ability to reach the tumor bed [53]. Hydrophilic polymers such as polyethylene glycol (PEG) and dextran are also commonly used to reduce opsonic adsorption of NPs to prolong their blood circulation. Amphiphilic block co-polymers can self-assemble into miscelles, i.e., NPs with a spherical structure with a hydrophobic core and hydrophilic surface. Adding imaging labels to polymeric NPs is similar to loading drug, i.e., imaging labels can be encapsulated/conjugated inside or outside of the polymeric micelles by directly labeling the polymer before or after micelle formation. For example, to label PLGA NPs for nuclear imaging, one route is to add ^99m^TcO_4_^−^ to PLGA polymers then perform NP assembly [54], hence encapsulating ^99m^TcO_4_^−^ in the NP core. While adding ^64^Cu labels to the amphiphilic PEG-b-PLA co-polymer, a chelator, NOTA, can be conjugated to the PEG component on co-polymers prior to radiolabeling, yielding ^64^Cu on the surface of the formed micelles [55].

An important subset of polymeric NPs is the dendrimer, which have tree-like structures. Dendrimers have a defined structure with the multiple surface reactive groups rendering dendrimers versatile for chemical modification [49], including the addition of conjugating drugs, imaging labels or targeting moieties [56,57,58]. For example, abundant amine groups of polyamidoamine (PAMAM) can be used to form linkage to doxorubicin via amide or hydrozone as coupling molecules [59], react with NHS-DOTA chelator for loading radioactive or paramagnetic meta ions [60], or conjugate with peptides [61] or antibodies [62] for active targeting. For these reasons, dendrimers are an important class of NP carriers currently in preclinical development and in clinical trials. To date, the most common dendrimers seen in preclinical and clinical development are PAMAM, poly(l-lysine) (PLL), polypropyleneimine (PEI) and peptide dendrimers [63]. The cationic charge of PAMAM and PEI dendrimers allow them to carry anionic drugs or genes (DNA/RNA) through electrostatic complexation, and are therefore important drug carriers. To endow imaging detectability, imaging labels are usually added to the surface of dendrimers. For example, when constructing radiolabeled PAMAM targeting to prostate cancer, Wojciech et al. first attached the DOTA chelators to the PAMAM, to allow subsequent ^64^Cu chelating [60]. A similar chelator-based approach can also be used to chelate Gd^3+^ ions to endow MRI-detectability to PAMAM [64]. As PAMAM and PEI are also commonly used to coat the surface of other NPs, image-detectability of the resultant nanoDDS can be generated using iron-oxide NPs as the core for MRI [65] or gold NPs as the core for CT [66].

#### 2.1.3. Metallic and Inorganic NPs

Metallic and inorganic NPs typically have a central core composed of materials that bestow unique optical, electric, fluorescent, or magnetic properties. Due to the potential toxicities associated with naked inorganic NPs, in many cases such inorganic NPs are coated with other biocompatible molecules, e.g., polymers such as PEG [67], chitosan [68] or dextran [69]. Inorganic NPs include mesoporous silica nanoparticles (MSNs), carbon dots (CDs), graphene quantum dots (GQDs), gold nanoparticles (AuNPs), and iron oxide nanoparticles (IONPs [23]). Compared to organic NPs, which are usually spherical, inorganic NPs can be manufactured as well-controlled physical structures and shapes. These include porous, core–shell, nanorods, and cubes, each of which can influence and be critical to their optical, thermal and magnetic-responsive properties.

One unique advantage of metallic and inorganic NPs is that some of them are inherently image-detectable. For example, some light-absorbing CDs, GQDs and AuNPs are imageable in fluorescence imaging or photoacoustic imaging. AuNPs are also CT contrast agents and IONPs are MRI contrast agents. Formulating those NPs as nanotheranostics obviates the need for adding extra image labels. If there is a need to enable multi-modal imaging by introducing another imaging label, metallic NPs, such AuNP, can be surface-activated to add imaging labels, while inorganic NPs, such as GQD, can form nanocomposites with Gd_2_O_3_, as an example, to gain MRI detectability [70]. These NPs can also form core–shell or porous structures to load imaging labels inside [71,72].

### 2.2. Targeting Moieties

Biological barriers are important components to consider when it comes to choosing a particular target in cancer. Commonly encountered barriers by nanoDDS are vascular, lymphatic and stromal structures, as well as clearance by the mononuclear phagocytic system (MPS) and kidneys [13]. The tumor vasculature and microenvironment both represent formidable barriers to nanoparticle uptake and accumulation; however, the unique properties of tumor vasculature and microenviroment can instead be exploited to facilitate drug delivery. The vasculature, which serves as a pipeline for intravenously administered NPs, can actually be targeted to enhance drug localization [73]. Neovasculature that has developed as a result of tumor growth heralds a unique array of molecules, which distinguish them from healthy tissues [74]. 

The key components of tumor stroma that limit particle diffusion are the dense extracellular matrix, the presence of phagocytic immune cells that trap NPs, and high interstitial pressure [75]. Despite the obstacles imposed by tumor stroma, unique characteristics of tumor stroma, such as low pH, relative hypoxia, and overexpressed oncoproteins, have also been pinpointed as potential drug targets to improve delivery efficiency [76]. For example, NPs have been equipped with acidic targeting peptides or hyaluronidase-based coatings specifically for targeting the microenvironment in pancreatic adenocarcinoma [77,78]. Such techniques exploit absolute characteristics of the microenvironment to maximize delivery of therapeutic drugs or imaging contrast agents to tumors while mitigating off-target accumulation [79,80]. 

### 2.3. Imaging Labels for Nanotheranostics

In this section, we will introduce commonly used methods for labeling NPs to endow detectability in different imaging modalities. Interestingly, as imaging labels themselves could introduce therapeutic effects to the particles, we will associate unique therapeutic properties of different imaging labels and their applications (Table 2). In addition, the intriguing properties of nanoDDS with inherent imaging detectability, e.g., iron oxide NPs, and their application will also be discussed. Recent years have seen tremendous preclinical development and several clinical applications of cancer nanotheranostics (Table 3). Here, we will provide an overview of the mechanism and NP labeling strategies for different imaging modalities, with an emphasis on prominent cancer nanotheranostics currently undergoing clinical testing.

#### 2.3.1. Radiolabels

Nuclear imaging modalities, including PET and SPECT are to date two of the most-used imaging modalities for nanoDDS owing to their capabilities for whole-body systemic assessment and the ability to quantify their signal. More importantly, in nuclear imaging nanoDDS can be detected in the microdose range (<1% of the therapeutic dose), which facilitates clinical translation [81]. The most commonly used positron-emitting radionuclides in clinical studies are β- and γ- emitters due to their manageable energy levels and long ranges [82]. Given the average circulating time of nanoDDS, radionuclides used for labeling NPs are usually those with long half-lives. Technetium-99 m is the most frequently utilized radionuclide because of its wide availability, low cost, and its long half-life (6 h), which permits an imaging window of up to 24 h. Isotopes of iodine and copper are also often used [83]. 

While various methods can be used to radiolabel NPs, one important consideration is that the synthesis of NPs has to be a lot shorter than the decay of radioisotopes to preserve their radiotracing functionality. Coordination chemistry is used to covalently label NPs with radioisotopes by forming a stable chelator-isotope bond in a short period. Chelators for metallic radioisotopes include esadentate acyclic chelators (e.g., ethylenediaminetetraacetic acid EDTA or DTPA), tetradentate acyclic chelators (e.g., PTMS), and macrocyclic chelators such as 1,4,7-triazacyclononane-N,N’,N’’-triacetic acid (NOTA) and 1,4,7,10-Tetraazacyclododecane-1,4,7,10-tetraacetic acid (DOTA) [84]. Notably, careful selection of chelators is critical as certain radioisotopes can also be effectively bound using specific chelators [85]. For example, macrocyclic chelators are generally considered to bond more strongly to metallic radioisotopes [86]. It has also been found that NOTA is more suitable for ^64^Cu labeling than DOTA [87,88,89].

NPs can be labeled by either attaching the chelators on the surface or by adding them to the NP payload. Encapsulation of radiolabels within liposomes can be achieved passively by a process of extrusion [90]. However, this approach requires fresh liposome preparation before imaging, which is labor-intensive, and suffers from a low loading efficiency <10%. Another mechanism for liposome radiolabeling is to use a lipophilic chelator to incorporate radioisotopes into the lipid layer [91]. A combined approach of “remote loading” has been devised to allow radioisotopes to diffuse through lipid layer of liposome encapsulating hydrophilic chelators, forming chelates “remotely” inside liposomes [92,93,94]. This approach has become increasingly popular due to its efficiency and has been adopted in clinical trials [95]. Other chelator-free radiolabeling approaches have been developed and applied in preclinical studies [96,97,98]. For example, a new approach has been derived to label nanographene with ^64^Cu based on transition metal–π electron interactions [97]. Rapid ^64^Cu and ^69^Ga labeling of quantum dots was also achieved through a cation exchange approach [99].

**Figure 2:**
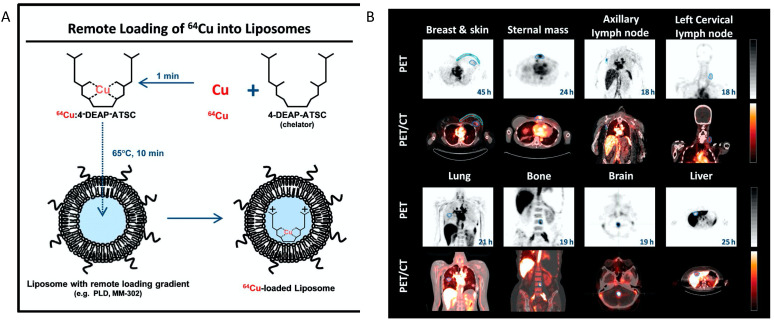
**The construction**^64^**Cu-MM-302 and its application in lesion detection in the phase I clinical trial (NCT01304797).** (**A**) Schematic depicting remote loading of ^64^Cu into liposomes using the novel gradient-loadable chelator 4-DEAP-ATSC. Heating liposomes above the lipid bilayer phase transition temperature facilitates transmembrane transport of unprotonated 4-DEAP-ATSC, which becomes protonated within the liposome and remains entrapped. (**B**) Representative PET and fused PET/CT images of ^64^Cu-MM-302 in lesions at different anatomic locations. Intensity scale bars represent deposition from 0 to 10%ID/kg (derived from SUVmedian). The regions of interest used to measure tumor deposition of ^64^Cu-MM-302 are shown in blue or turquoise outlines. ^64^Cu-MM-302 uptake was detected at above muscle background level in lesions of various anatomic locations that are common for HER2-positive metastatic diseases. Figures are adapted based on Refs. [95,101] with permissions. Copyright 2017 American Association for Cancer Research.

One prominent clinical application of radiolabeled nanotheranostics is of radiolabeled liposomes. In the clinical trial of a formulation of PEGylated liposomal doxorubicin targeted to human epidermal growth factor receptor 2 (HER2) (NCT01304797) named MM-302 [95], 19 patients with metastatic breast cancer were selected for imaging study using Cu-64 labeled MM-302, [^64^Cu]MM-302. This radiolabeled liposome was surface-functionalized with an anti-HER2 scFV-PEG-DSPE, which inserts into liposome bilayer [100]. ^64^Cu chelated by a novel chelator 4-DEAP-ATSC was loaded by gradient into liposomes [101] (Figure 2). Directed at testing whether HER2-targeting increases the amount of drug accumulating at the metastases, and further correlating with the efficacy of trastuzumab treatment (a clinically approved anti-HER2 monoclonal antibody), [^64^Cu]MM-302 at a target dose of 400 MBq per patient was given and PET imaging at this 24 h showed that [^64^Cu]MM-302 remained in the circulation for over 24 h, with the liver and spleen being the major organs of NP uptake. Importantly, it found that high ^64^Cu-MM-302 deposition in tumors was associated with more favorable treatment outcomes. This study exemplifies the use of imaging probes for patient stratification and outcome prediction.

Because of their high energy levels and ionizing potential, α-emitters are usually included as the payload of an NP rather than attached to the particle surface. Their β- and γ-emitter counterparts can help define the dose and rate at which the radionuclides are delivered to tumor versus normal tissues before α particle therapy due to similar pharmacokinetics. One such example is the use of SPECT/PET imaging with ^123^I/^124^I-labeled agents before ^131^I-based radionuclide therapy [102,103,104]. These identical diagnostic/therapeutic pairs enable a theranostic regime for reliable delineation of biodistribution, target site accumulation, and prediction of responsive tumors. 

Despite the fact that radiolabeled liposomes constitute an overwhelming majority of radiolabeled NPs in clinical studies, recent development of other types of radiolabeled NPs, including inorganic NPs, e.g., silicon NPs [105,106], and polymeric NPs including cellulose [107] and chitosan NPs [108], are gaining momentum. In studies performed by Cai et al., a novel type of ultrasmall porous silica nanoparticles (UPSN) (size ~15 nm) were labeled with isotopic pair yttrium-90/86 (^90/86^Y, with the high energy β-emitter ^90^Y being used for therapy and low energy emitter ^86^Y for imaging) through the DOTA chelators. The smaller size of these radiolabeled UPSNs led to enhanced in vivo pharmacokinetic behaviors, achieving an astonishingly high tumor accumulation (12% ID/g), long blood circulation, and greater evasion from the RES system. In mouse models of breast cancer, theranostic NPs enabled both sensitive detection of tumors (with 10.4 ± 0.8% ID/g uptake of ^86^Y-DOTA-UPSN in tumor sites), and efficient treatment monitoring and tumor retardation (~30% tumor regression) after injecting ~5.5 MBq ^90^Y-DOTA-UPSN [106].

#### 2.3.2. Magnetic Resonance Imaging Labels

Most MRI labels generate contrast by indirectly affecting neighboring water molecules. The movement of these water molecules is detected and translated into an MRI image which is based on relative tissue water content. Paramagnetic labels, which generate movement through weak magnetic forces, include Manganese (Mn^2+^; Mn) as well as lanthanide metal ions such as gadolinium (Gd^3+^; Gd). These paramagnetic labels generate positive (brightening) signals in MRI images. Paramagnetic metal ions are used in chelated form since the accumulation of the naked ions in tissues typically induces toxicity [109]. Chelators, such as DTPA and DOTA, are also used in constructing metal ion MRI labels to confer thermodynamic and kinetic stability. The ability of MRI labels to generate image contrast is measured by its effects on shortening water T1 and T2 relaxation times, metrics termed r1 and r2 relaxivity, respectively. Current clinical Gd-based contrast agents have an r1 relaxivity of 3–4 s^−1^mM^−1^ at field strengths of 0.5 Tesla and 37 °C. Much scientific effort has been devoted to improving relaxivities of the paramagnetic agents to enhance detection sensitivity and lower contrast agent doses. While small-molecular targeted agents usually contain one or several paramagnetic ion chelates per molecule, demanding an abundant level of their molecular targets, targeted NPs that encompass hundreds or thousands of paramagnetic ion chelates per particle can enhance detection sensitivity by increasing chelate-to-target ratios.

**Figure 3:**
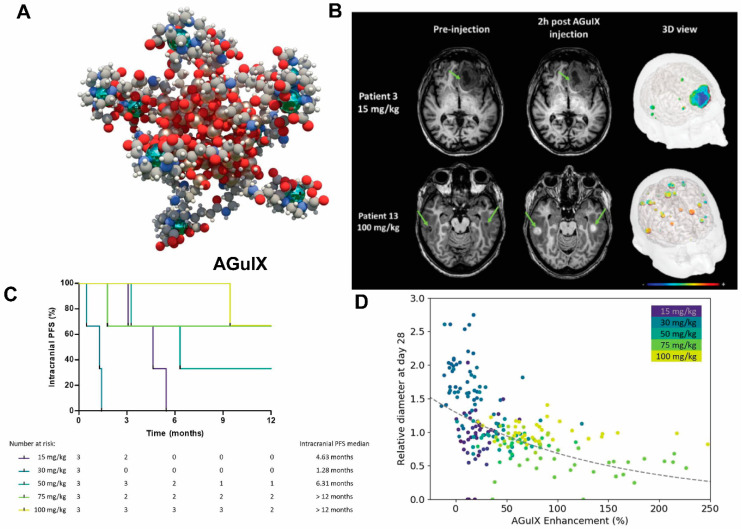
**AGuIX as radiosynthesizer and MRI contrast-enhancing NPs in the phase I clinical trial (NANO-RAD trial).** (**A**) Schematic representation of AGuIX. Gadolinium ions are chelated by 1,4,7,10-tetraazacyclododecane-1,4,7,10-tetraacetic acid derivatives. Polysiloxane core (Si, metallic grey; O, red; H, white; C, grey; N, blue) is surrounded by covalently grafted chelates of gadolinium (Gd, metallic green). (**B**) AGuIX contrast-enhanced MRI at 2 h in brain metastases of 2 patients with lung cancer following intravenous AGuIX administration at 15 and 100 mg/kg, respectively. T1-weighted MRI images were obtained without injection of contrast agent before and at 2 h after a single AGuIX intravenous administration at the indicated concentration. Green arrows are pointing highlighted metastases. The 3-D vizualization of entire brain with specific contrast enhancement into metastases was obtained from T1-weighted MRI mapping. (**C**) Intracranial progression-free survival (PFS) of multiple patients with brain metastases treated with a combination of whole-brain radiotherapy (WBRT) and different dose levels of intravenous AGuIX. The color of survival curves corresponds to different AGuIX doses. (**D**) Correlation between change in size of brain metastases and AGuIX signal variation. Correlation of measured metastasis sizes for patients with brain metastases and treated with whole brain radiotherapy and different AGuIX doses. Points colored according to patient number and administrated dose with darker colors corresponding to lower AGuIX doses. Metastasis diameter at 28 days normalized to diameter at Day 0 (V28/V0) as a function of AGuIX enhancement (points) compared with predicted trend (dashed line), showing good agreement and dependence of metastasis evolution on AGuIX uptake. AGuIX, Activation and Guidance of Irradiation by X-Ray. Figures were adapted based on Refs. [111,113] with permissions.

One prominent type of paramagnetic NPs seen in clinical trials is AGuIX (Activation and Guidance of Irradiation by X-ray), which are sub-5 nm NPs composed of a polysiloxane matrix with gadolinium cyclic chelates covalently grafted on the inorganic matrix [110,111] (Figure 3). Gd retention by brain tumor cells following AGuIX injection means AGuIX NPs have high radiosensitizing properties [112] together with excellent positive MRI contrast (r1 = 8.9 mM^−1^s^−1^ per Gd at 3 Tesla) [111], making them powerful nanotheranostic agents. Phase-I clinical trials, regulated by the French Agence Nationale de Sécurité du Médicament et des produits de santé, have been conducted (NANO-RAD trial, NCT02820454 [78]). In this trial, patients with brain metastases were given intravenous injection of escalating doses of AGuIX (15, 30, 50, 75, or 100 mg/kg b.w.) on the day of initiation of whole-brain radiation therapy (30 Gy in 10 fractions). This study demonstrated no dose-limiting toxic effects up to AGuIX 100 mg/kg, with a mean half-life of AGuIX shown to be 1.3 h at all doses. Efficiency and persistence of AGuIX contrast enhancement were observed in brain metastases from patients with primary colon cancer, melanoma, lung, and breast cancers. More importantly, 13 of 14 evaluable patients had improved clinical outcomes evidenced by either stabilized or reduced tumor volume. A significant correlation was found between MRI contrast enhancement and tumor response, implicating a radiosensitizing effect. From the perspective of image-guided therapy, this study provides strong suggestion that imaging can serve as a non-invasive predictor of cancer treatment outcomes [113]. The phase II trial of AGuIX is underway to expand the protocol to multiple centers and 100 patients. It is worth noting that AGuIX mainly relies on EPR effect for tumor homing, and suffers from a low tumor residence time and strong off-target effect. A newer version of AGuIX, which includes porphyrin as an extra photosensitizer and is modified with peptides targeted to neuropilin-1 (NRP-1), a transmembrane receptor abundantly overexpressed in the tumor vascular system [114], is undergoing preclinical testing.

An interesting application of Gd-based NPs is in photodynamic therapy (PDT). Since Gd has a high ^1^O_2_ quantum yield upon light irradiation and several studies show the high-relaxivity, Gd-encapsulating NPs such as Gd-graphene carbon [115] and gadofullerenes can serve as photosensitizing agents. Upon activation under light of a specific wavelength, these particles trigger a cascade of tumor-damaging photochemical and photobiologic reactions, such as generating reactive oxygen species (ROS). Guan et al. prepared a β-alanine(Ala)-modified gadofullerene (Gd@C_82_-Ala, diameter = 130 nm) that shortens the light interval between Gd-Alanine under light irradiation and induces malignant tumor cell and vascular disruption. This study showed that following Gd@C_82_-Ala administration, localized treatment with white light irradiation for 30 min led to significant retardation of tumor growth accompanied by increased blood vessel porosity and immune cell recruitment [116]. Gadofullerene has also been used to treat melanoma [117]. These studies, together with the capability of Gd-encapsulatig NPs as sensitive MRI probes with a high transmetallation stability [118], indicate great potential of Gd-encapsulatig NPs as nanotheranostics for cancer.

Beside paramagnetic ion chelates, superparamagnetic NPs, majorly iron oxide (Fe_3_O_4_) NPs or magnetite, are frequently used by themselves or after being incorporated into another NP matrix. In fact, iron oxide NPs constitute a large portion of clinically approved NPs, e.g., Feraheme, a dextran-coated iron oxide particle for treating anemia, and therefore has been the focus of NP research. The size of iron oxide particles may range from several nanometers, i.e., super-small iron oxide nanoparticles (SPIONs), to micron-sized nanoparticles (IONs), with intrinsic r1 and r2 relaxivities scaling with the size and coating composition. Larger IONs (diameter >10 nm) predominantly generate T2/T2* contrasts, which manifest as “darkening” contrasts in images. Ultrasmall IONs (USPION, diameter <10 nm) can also generate T1 contrasts, and the composition of USPIONs can be tuned, e.g., by adding gadolinium [119], to exhibit both T1 and T2 contrasts (also dubbed as dual-contrast agents). Overall, SPIONs are favored as a cargo in nanoDDS due to their small size, unless IONs themselves serve as the drug carriers. 

IONs may also enhance therapeutic efficiencies of nanoDDS. The thermal effects of iron oxide NPs under an alternating magnetic field (AMF) can be used for cancer therapy [120,121]. By targeting iron oxide NPs to cancer cells, magnetic hyperthermia treatment (MHT) can induce specific cancer cell death as the tumor environment temperatures increase to >41 °C. The specific absorption rate (the rate of energy absorbed per unit mass under radio frequency) [120] increases with particle size, and therefore most studies use IONs of 20–50 nm [120]. In the study by Ishimura et al. [122], folic acid-conjugated PEG-coated SPION clusters were constructed as targeted nanotheranostics for MRI and MHT. The clustering of SPIONs not only prolonged blood circulation, but also enhanced relaxivity and SAR. It was shown that after intravenous injection, the clusters showed significant MRI contrast enhancement in breast cancer tissues and exerted high magnetic hyperthermia effect (*f* = 230 kHz, *H* = 8 kA/m). Additionally, the magnetism of magnetite could also be exploited to create another driving force for targeted delivery. For example, magnetically labeled nanoDDS can be navigated to cancerous regions under an external magnetic field–a technique termed magnetic targeting, which has been shown to improve efficacy in preclinical models [123,124,125]. Active targeting of NPs can also be combined with magnetic targeting to enhance chemotherapy drug delivery. 

**Figure 4:**
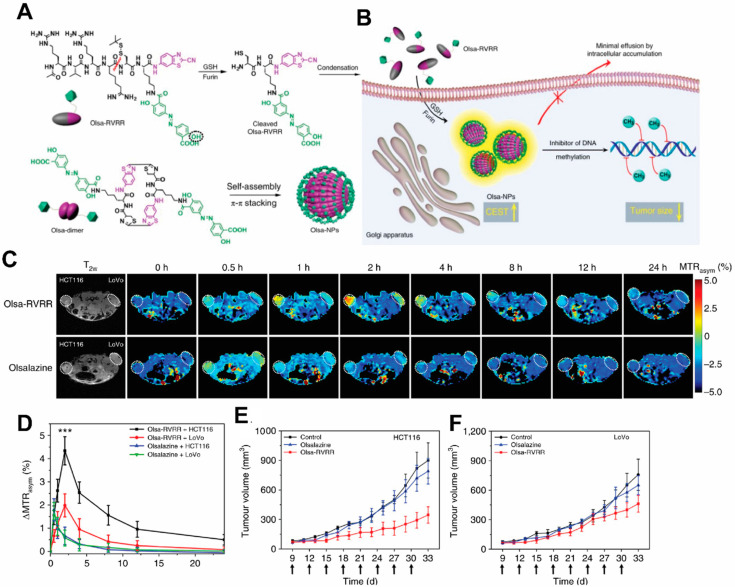
**Schematic illustration for the formation of Olsa-NPs by furin-mediated intracellular reduction and condensation of Olsa-RVRR, resulting in enhanced CEST signal and tumor treatment efficacy.** (**A**) Self-assembly of Olsa-RVRR into Olsa-NPs through a series of steps. Red line indicates the site of furin cleavage, and the circled hydroxyl group indicates the exchangeable hydroxyl proton that provides OlsaCEST signal at 9.8 ppm from the water frequency. (**B**) After Olsa-RVRR enters the cytoplasm of high furin-expressing cells (the HCT116 colon cancer cells in this study), it undergoes reduction by GSH and cleavage of the peptide by furin near the Golgi complex where cleaved Olsa-RVRR is generated. Amphiphilic oligomers (mostly dimers) are then formed from the click reaction between two cleaved Olsa-RVRR molecules, followed by self-assembly into Olsa-NPs as a result of intermolecular π-π stacking. The intracellular accumulation of Olsa-NPs then serves as a reservoir of Olsa molecule-enhancing CEST contrast and inhibiting DNA methylation for tumor therapy. (**C**,**D**) Dynamic T2-weighted (T_2w_) and OlsaCEST serial MRI of tumor-bearing mice after intravenous injection of 0.2 mmol kg^−1^ Olsa-RVRR or Olsa (left, HCT116; right, LoVo colon cancer cells). Time course MTR_asym_ maps (**C**) and MTR_asym_ OlsaCEST signal (**D**) for tumors after background correction by the subtraction of the MTR_asym_ value at 0 h. Data are shown as mean ± s.d. for *n* = 4 mice; one-way ANOVA, followed by Dunnett’s post hoc test; ***: *p* <  0.001 versus all other groups. (**E**,**F**) Anti-tumor effects of Olsa and Olsa-RVRR for HCT116 (**E**) and LoVo (**F**) tumors. Arrows indicate time points of repeated drug administration (every 3 d × 8) after tumor cell injection. Data are shown as mean ± s.d. (*n* = 4 mice). The figure is adapted with permission based on Ref. [126]. Copyright 2019 Springer Nature.

It would be ideal to combine detection properties of both the NPs and the drug without additional labeling. Recent development of Chemical Exchange Saturation Transfer (CEST) MRI gives a glimpse of this possibility. This imaging modality offers the potential to detect diamagnetic compounds, i.e., compounds which do not possess metallic labels, which encompasses most drugs and organic NP matrices. In a recent study by Yuan et al., a self-assembly enzyme-responsive NP was constructed for image-guided cancer therapy (Figure 4). The building blocks of the NPs are an anticancer agent olsalazine (Olsa) conjugated to the cell-penetrating peptide RVRR. Under enzymic reaction by furin, these NPs self-assemble into large intracellular NPs [126]. Both the NPs and their constituent peptide components are readily detected with CEST MRI by virtue of exchangeable Olsa hydroxyl protons. In vivo studies showed that the NPs result in generation of a 6.5- fold increase in tumor CEST contrasts and 5.2-fold increase in anti-tumor therapeutic effect in colon cancer, compared to Olsa treatment alone. Besides Olsa, this effect is thought to apply to some other chemotherapy drugs including gemcitabine [127,128] and melphalan [129]. Readers are referred to reviews on CEST-detectable nanoDDS for more details on the topic [130,131].

#### 2.3.3. Ultrasound Labels

**Figure 5:**
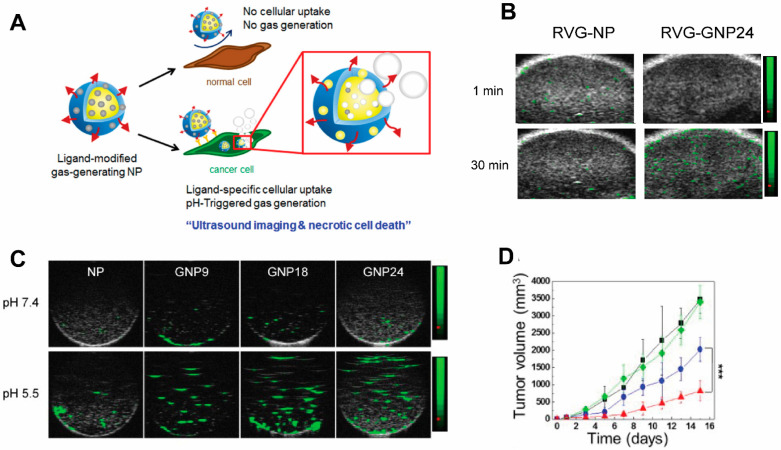
**A targeted gas-generating nanotheranostic particle for ultrasound-guided treatment of neuroblastoma.** (**A**) Schematic illustration of ligand-modified gas-generating nanoparticles for cancer-specific cellular uptake and pH-triggered gas generation. PLG nanoparticles loaded with fine-grained calcium carbonate provide theranostic functionality for cancer detection and treatment. pH change triggered carbon dioxide gas generation and these bubbles enabled simultaneous US imaging and necrosis of cancer without using conventional contrast or anti-cancer agents. (**B**) In vitro ultrasound signals of non-gas-generating NP and gas-generating nanoparticles (GNP9, GNP18, and GNP24) under neutral and acidic conditions ([GNP] = 10 mg/mL). (**C**) In vivo ultrasound imaging of tumors after intravenous injection of RVG-NP and RVG-GNP24 into a tumor-bearing mouse model (25 mg/kg, polymer/mouse at 1 and 30 min). (**D**) Changes in tumor volume of mice treated with saline (black diamond), RVG-NP (green square), RVG-GNP9 (blue circle), and RVG-GNP24 (red triangle) (10 mg/kg polymer/mouse and 20 mg/kg docetaxel/mouse; five daily intravenous injections; *** *p* < 0.001). Figures are adapted based on Ref. [144] with permission. Copyright 2016 Elsevier.

Ultrasound (US) is one of the earliest-employed diagnostic imaging tools. Its application in cancer offers unique benefits of both portability and real-time depiction of tumors [132]. US has also been employed as a remarkable therapeutic tool by locally inducing drug release from carriers [133,134,135] to perform thermal ablation therapies, i.e., high-intensity focused ultrasound (HIFU) [136], among other applications [137]. A major class of ultrasound contrast agents are gas-filled nano-/micro-bubbles and liposomes with high echogenicity [138], i.e., the ability to reflect the ultrasound waves, thus generating enhanced sonogram. Under focused US, which induces oscillation of the gas bubbles in a fluid (a mechanical phenomenon termed inertial cavitation) [139], gas bubbles grow unstable and subsequently collapse during compression under the inertia of the surrounding fluid. Hence, US can be used to enhance delivery efficiency of therapeutic agents to the tumor beyond the intrinsic targeting of NPs [140]. In HIFU, gas-containing NPs intensify the thermal response in target sites to enhance specific thermal ablation and decrease damage to normal tissues. The commercial organic microbubbles or liposomes in use for US imaging are lipid-coated perfluoropropane (phase transition temperature of 56 °C) microbubbles [141], namely Levovist, Sonovue, and Optison, which undergo an instant phase transition into echogenic gas bubbles. Their micrometer size and limited longevity due to premature rupture make them undesirable as drug delivery systems and HIFU agents, and they have only been used clinically as US contrast agents thus far. Efforts have been devoted to developing particles encapsulating other phase-transition materials including perfluorohexane (PFH, phase transition temperature of 56 °C) [142] and perfluoropetane (PFP, phase transition temperature of 29 °C) [143], for a controllable phase transition, and the use of NPs for a higher targeting to tumors. Another interesting study also encapsulated calcium carbonate using poly(d,l-lactide-*co*-glycolide)(PLG) to construct a NP for treating neuroblastoma (Figure 5). In this work, the gas-generating NPs (GNPs) were modified with a rabies virus glycoprotein (RVG) peptide targeted to the nicotinic acetylcholine receptor (nAChR) abundantly expressed in neuroblastoma. At the tumor’s low-pH microenvironment, these NP are triggered by the pH change and generate carbon dioxide bubbles that imposes physical shock to cancer cells, simultaneously enhancing US contrasts [144]. This allows for the verification of the accumulation of NPs within tumors by US imaging. Despite not carrying additional chemotherapy drugs, thereby obviating side effects associated with conventional chemotherapy, necrotic cell death induced by the GNPs led to markedly retarded tumor growth.

Besides micro/nano-bubbles and polymeric nanoparticles, recent studies have also demonstrated the potential of inorganic NPs as US-detectable nanotheranostics. For example, Zhang et al. constructed a type of core–shell nitrogen-doped graphene quantum dot (N-GQD) coated with the water-splitting agent carbon nitride (C_3_N_4_) [145]. The NPs were also decorated with integrin α_v_β_3_-targeted RGD peptides to facilitate active targeting to tumor. Upon irradiation with laser, the C_3_N_4_ layer splits endogenous H_2_O molecules and induces generation a substantial amount of O_2_ bubbles, and hence contribute to enhanced echogenic reflectivity in tumor. The elevated oxygen level also alleviates tumor hypoxia through oxygenation. Besides, the fluorescent quantum dots in the core of these particles also contributes to its detectability in fluorescence imaging and infrared thermal imaging, as well as role as a photothermal agent. C_3_N_4_ also serves as PDT agent to synergize with other therapeutic functionalities of the NPs. Similarly, other oxygen-generating photosensitizers have been used to construct NPs with US detectability [146,147,148]. For example, Gao et al. constructed indocyanine green (ICG) modified hyaluronic acid nanoparticle encapsulating manganese dioxide (MnO_2_) NPs, which are photosensitizers that react with endogenous H_2_O_2_ to generate oxygen bubbles. This study demonstrated that tumor accumulation of the NPs led to 2.25 times higher oxygen contents in tumor as confirmed by ultrasound imaging. This nanotheranostic system was also demonstrated to inhibit growth of squamous cell carcinoma in mice and improve survival through PDT [147].

#### 2.3.4. Optoacoustic Labels

OAI, also known as photoacoustic imaging, is an emerging modality based on the “light-in sound-out” principle, which has garnered increasing attention. In OAI, NPs can be loaded with small-molecule organic dyes with high photothermic conversion efficiency, such as IR780 or ICG, to become imageable. Several near-infrared light (NIR)-absorbing NPs, such as gold nanoparticles, iron oxide particles, semiconductor NPs, can also be used in OAI to illustrate the biodistribution of injected NPs [149,150]. However, not all OAI agents are created equal, and the conversion efficiency of optical energy into pressure waves is dependent on several factors. Controlling the geometry, composition, coatings, and solvents around plasmonic nanostructures can each help to generate the optimum OA signal [151].

Organic NIR dyes are common OAI contrast agents for NP labeling, as NIR dyes have a high extinction coefficient and low quantum yield, with an ideal spectral window (NIR-I: 650–950 nm or NIR-II: 1000–1700 nm) that overlaps negligibly with the biological background. The absorption wavelengths in this range also allows the excitation light to penetrate as deep as a few centimeters into the tissue. Common OAI dyes include squaraine, semicyanine, pentamethine cyanine, heptamethine cyanine, porphyrin, perylene-diimide, aza-BODIPY, and benzobisthiadiazole [152,153].

**Figure 6:**
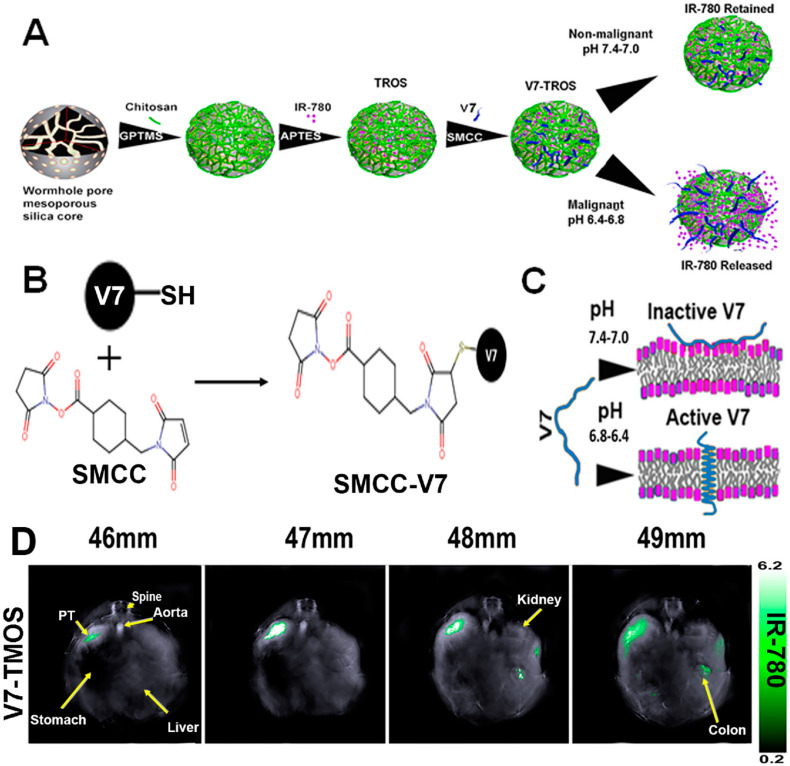
**Construction of the low-pH-targeted MSN NP for photoacoustic imaging of pancreatic ductal adenocarcinoma.** (**A**) Schematic Illustration of the Components and Formation of V7-TROS NPs, including V7-TMOS, V7-TEOS and V7-TPOS. (**B**) Conjugation Chemistry of SMCC to the Cysteine Residue on the V7 Peptide, and (**C**) the Activation Mechanism of the V7 Peptide in Acidic Environments. (**D**) Biodistribution of V7-TMOS in axial slices showing accumulation within the tumor, kidney, liver, and spleen Figures were adapted based Refs. [77,154] with permissions. Copyright 2021 American Chemical Society.

A large class of OAI nanotheranostics are NPs that encapsulate NIR dyes. MSNs are a class of NPs extensively studied for OAI-guided drug delivery. In the work by our group and others, MSNs with various sizes and pore structures have been developed. In work by MacCuaig et al., MSNs with wormhole pores were used to load IR780 OAI dye and chemotherapy drug paclitaxel. The asymmetric morphology of wormhole pores was to provide a higher surface area for increased loading capacity and slower cargo release. The particle also has a chitosan coating as the gate keeper, as chitosan shrink at physiological pH to entrap the cargo but expands at low pH for cargo release. To endow targeting capabilities, a pH-low-insertion peptide (pHLP) V7 was conjugated to the NP surface so that the NPs home to low-pH tumor microenvironment, where V7 peptide facilitate cellular uptake of the NPs. The resultant NP, named V7-TROS, was demonstrated to efficiently translocate into the cytoplasmic compartment for the release of the IR780 dye and paclitaxel, leading to enhanced tumor contrast and anti-neoplastic efficacy on ovarian cancer [154]. The low-pH targeted nanotheranostics were also found to enhance tumor detection using OAI and cargo uptake in orthotopic pancreatic cancer [78] and triple-negative breast cancers [155]. With the guidance of multiple spectral optoacoustic tomography (MSOT), the study also showed that active targeting outperforms NP size in facilitating tumor-specific uptake (Figure 6). 

Gold nanostructures have been widely used for OAI because of their unique optical and physicochemical properties. Upon illumination by a specific light, the gold (Au) nanostructures generate surface plasmons that leads to the absorption of the light, followed by conversion of the optical energy to heat, pressure, and then acoustic wave. Interestingly, AuNPs with different shapes have shown different OAI characteristics. For example, Au nanorods with rationally tuned shape, e.g., high aspect ratio, can have an absorption region in the NIR region [156], but spherical AuNPs only have absorption in the visible region, making it unsuitable for in deep-tissue imaging. Besides, semiconducting nanoparticles (or quantum dots, QDs) have also been used as OAI nanotheranostics as their optical absorption properties can be easily tuned to the NIR region by choosing the appropriate particle size [157,158]. 

The photothermal phenomena used in OAI can also be extended to therapeutic applications. In photothermal therapy (PTT), cancer-localizing high-efficiency photothermal NPs can generate heat under NIR light irradiation. In a recent study, Dai et al. employed the photoacoustic and photothermal effect of Au nanorods to construct a chitosan/gold NP that load the tumor suppressor p63 plasmid DNA for breast cancer gene therapy [159]. This study demonstrated the feasibility of OAI-guided synergistic PTT/gene therapy for malignant breast tumors, where PTT enhances gene therapy. It is worth noting that some OAI dyes are also PDT agents, e.g., porphyrin [160], and therefore can be used for simultaneous OAI-guided PTT/PDT therapy to maximize anti-cancer efficacies.

#### 2.3.5. Computed Tomography Labels

**Figure 7:**
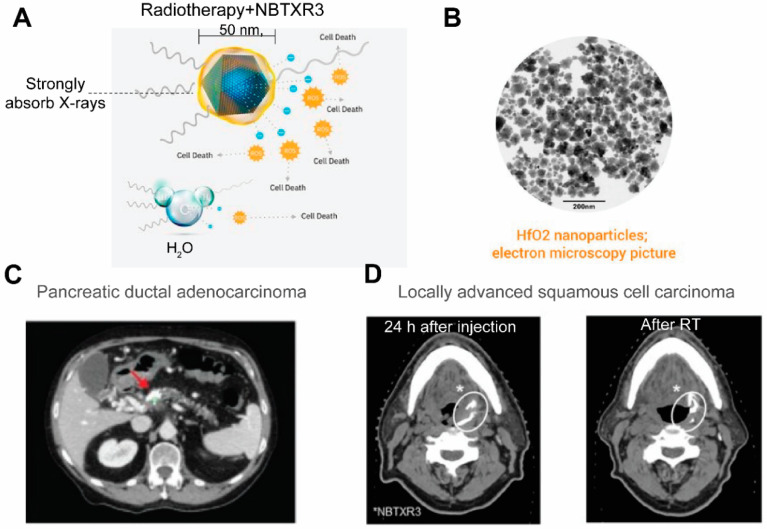
**NBTXR3 as radioenhancers that are trackable by CT.** (**A**) An illustration of the composition of NBTXR3 and its radioenhancing function. NBTXR3 consists of the HfO2 crystalline core (blue) and phosphate coating (yellow). Upon ionizing radiation, HfO2 induces the generation of a substantial amount of electrons that create more energy deposition in tumor than water molecules, hence promoting cancer cell death. Source: twitter@Nanobiotix. (**B**) Electron microscopy image of NBTXR3. Source: https://www.sharepitch.com/healthcare/archives/05-2018, accessed on 22 April 2022. (**C**) Radiopaque NBTXR3 visualized on simulation CT image of a patient with pancreatic ductal adenocarcinoma (red arrow: tumor). Adapted based on Ref. [165]. (**D**) CT scans showing intratumoral localisation of NBTXR3 at 24 h after injection and after radiotherapy (RT) in a patient with locally advanced squamous cell carcinoma. *: position of NBTXR3 accumulation.Adapted based on Ref. [166].

Currently CT stands as the leading radiologic method for biomedical imaging. The contrast agents for CT are X-ray attenuating agents, including iodine and high atomic metallic nanoparticles such as gold [161,162] and bismuch NPs [163,164]. As a large dose is required to generating CT contrasts, nanotheranostics designed for CT are relatively rarer compared to those designed for other imaging modalities. One prominent example of CT nanotheranostics is NBTXR3, which are 50 nm Hafnium oxide (HfO_2_) crystalline NPs functionalized with anionic phosphate coating (Figure 7A,B). NBTXR3 NPs act as radioenhancers to increase energy deposition in tumor during radiotherapy and their CT contrasts allows visualization of their accumulation in tumors. There are several clinical trials that investigate the efficacy of NBTXR3 in an array of cancer types (Table 3). In trials on pancreatic ductal adenocarcinoma (Figure 7C) [165] and locally advanced squamous cell carcinoma (Figure 7D) [166], the accumulation and retention of NBTXR3 in tumor were visualized by CT, which is valuable in evaluating the biodistribution of injected NPs and confirming persistence of NBTXR3 during the entire duration of radiotherapy.

It is worth noting that since metallic nanoparticles such as AuNP may also exhibit fluorescence, photoacoustic and photothermal properties, the ability of these NPs to generate CT contrast is frequently exploited to enhance their benefit as multimodal platforms. Notably, clinical iodinated CT agents, such as iodixanol [130] and iopamidol [167], also have CEST MRI detectability. An interesting study has constructed liposomes encapsulating iodixanol for tracking liposome intratumor accumulation using an MRI/CT dual-modality regime [168]. This approach may be easily adapted for other FDA-approved iodinated agents, using their clinical iodine dose, and thus has great translational potential.

## 3. Conclusions and Future Directions

Imaging is one of the tools best poised to enable tailoring of cancer therapies. The field of image-guided nanotheranostics has the potential to harness the precision of modern imaging techniques and couple this with the sophistication of nanomedicine. Drug delivery can be guided and tracked by the chosen imaging modality, which can then be used to confirm molecular targeting and dictate drug release. Imaging may also be used to visualize drug distribution and elimination, resulting in important insights into patient selection, drug pharmacodynamics and pharmacokinetics, all of which can be used to further tailor cancer therapies on both the individual and population level. Hence, imaging can provide additional information that biopsy and blood sampling cannot, helping to achieve the full potential of nanoDDS. Since their inception, NPs have not been restricted to act only as drug delivery vehicles—the use of NPs to improve imaging diagnosis has been highly anticipated. The utility of NP based imaging in an ideal future state would not be restricted to diagnosis, but also to therapeutic interventions such as cancer surgery. As a specific example, NP imaging platforms could be used as a supplement to intraoperative frozen sections to ensure that all microscopic cancer cells have been removed and that the margins are clear.

Despite this, the development of NanoDDS has largely outpaced the clinical development of NPs for imaging. Precision medicine instead gives a unique opportunity for combined therapeutic and diagnostic purpose for NPs, i.e., nanotheranostics. The past two years have seen a dramatic change in the nanomedicine landscape, driven by the worldwide adoption of the Moderna and Pfizer-BioNTech COVID-19 lipid nanoparticle mRNA vaccines [169]. Such expansion of the clinical use of NPs has resulted in an explosive increase in the number of clinical trials testing drug-encapsulated NPs (>35 new NP technologies, associated with >55 new trials) [169]. The continuous technological advancement surrounding both clinical imaging and nanotheranostics makes this the perfect match, as both areas are expected to see exponential growth in the coming years. The development of both of these fields will undoubtedly result in more refined designs for image-guided cancer therapies, potentially even distinguishing between closely associated diseases, and truly cement the promise of precision medicine. 

However, the concept of image-guided nanotheranostics has some limitations at this stage. Although being extensively used in clinical practice, each imaging modality covered in this review has both advantages and disadvantages. Nuclear medicine imaging has the highest sensitivity (pM range) and quantitative property, but suffers from a poor spatial resolution (mm range) [170]; CT excels at rapid image acquisition and facile three-dimensional (3D) reconstruction, but has limited resolution in soft tissues [171]; MRI has a high spatial resolution and excellent soft-tissue contrast with the versatility to provide information regarding tissue metabolism and perfusion. However, MRI suffers from lower sensitivity and hence requires a higher contrast agent dose to achieve necessary resolution [172], although recent advances in nanotheranostics have enhanced the per particle and per metal ion relaxivity [173,174]. It is also challenging to perform whole-body assessment using MRI and US, and therefore it is foreseeable nuclear imaging will remain as the quantitative whole-body approach in the near future. For NP-based imaging to be practical for use in the operating room or other interventional settings, it would be necessary to develop novel handheld detection devices and/or probes that are ergonomic and practical for use and sterilization, where US and OAI possess unique advantages. While no single modality is currently capable of obtaining all desired information, the combination of two or more imaging modalities, also called multimodal imaging, could offer synergistic effects [175]. The goal and future of diagnostic and imaging theranostics will, therefore, require a clear idea of what questions are being asked prior to choice of imaging platform. However, with greater sensitivity and specificity, diagnostics will allow greater expansion in therapeutics that allow for disruption of maladapted processes in each patient, i.e., tailored therapy. 

It should also be noted that while the field of image-guided drug delivery develops, the pharmacokinetics of these drugs may only reflect the behaviors of imaging labels or the labeled component of NPs. Attaching the imaging labels to the particle surface, although frequently used, circumvents the risk of altering pharmacodynamics of the drug itself, but may overshadow the drug’s natural behavior. Image-guided release and confirmatory tracking of the drug is reliant on their remaining paired throughout. For example, the biodistribution of radiolabelled liposomes is highly affected by the position of the radiocomplex. In a study by Tessa et al., liposomes that contain ^111^In in the lipid bilayer demonstrated significantly higher liver uptake than those that encapsulate. ^111^In [176], labeling the drug itself could provide information on the localization of the drug but involves chemical modification of the drug. Co-loading of imaging agents with drugs with NPs is another option, but the release profile of the imaging agent and the drug may still differ. The use of drug and NPs with inherent imaging detectability, such as AuNP, ION particle and CEST MRI-detectable NPs and drugs, could obviate the need to add imaging labels and thereby boasts unique advantages.

This review is not intended to provide a comprehensive list of imaging labels and NPs for image-guided therapy. Promising new imaging labels—such as 19F for MRI [177]; new imaging modalities such as magnetic particle imaging (MPI) [178] and Ramen spectroscopy [179]; as well as emerging biologic/biomimetic NPs, such as extracellular vehicles [180], the iron-storage protein Ferritin [181], and cell membrane-based NPs [182]—have recently entered the arena and are likely be added to the toolbox for cancer nanotheranostics. It can be envisioned that the explosive development of NPs in recent years will continue.

## Figures and Tables

**Table 1:** Clinical organic and inorganic nanoparticles designed for cancer imaging or therapy.

	NP Type	Drugs Loaded	Clinical Stage	Reference
**Organic NPs**	Liposome	Irinotecan, Doxorubicin, mRNAs	FDA-approved	[22]
	Polymeric Nanoparticles	Paclitaxel, Gemcytabine, Doxorubicin, Platinum	FDA-approved	[23]
	Dendrimer	Camptothecin, Doxorubicin	Preclinical	[24,25]
**Metallic and inorganic NPs**	Mesoporous Silica Nanoparticle (MSN)	Gemcitabine, Paclitaxel or Irinotecan	Preclinical	[25,26]
	Carbon Dots (CDs)	Gemcitabine or Cyanine 7	Preclinical	[25,27,28]
	Graphene Quantum Dots (CQDs)	Gemcitabine	Preclinical	[29]
	Gold Nanoparticles (AuNPs)	Gemcitabine and miR-21 inhibitor or Cetuximab	Clinical Phase I/II	[29,30,31,32]
	Iron Oxide Nanoparticles (IONPs)	Gemcitabine or Doxorubicin or Imiquimod	FDA-approved	[33,34,35,36,37]

**Table 2:** Imaging labels and their potential therapeutic properties.

	Imaging Labels	Therapeutic Properties
Nuclear Imaging	Radioactive Isotopes	Radiotherapy
**MRI**	Lanthanide metal ions, including Gd^2+^, Mn^2+^	Radiosensitizing
	Iron oxide nanoparticles	Photodynamic therapy
		Magnetic hyperthermia treatment
	Labile protons	Magnetic targeting
**Ultrasound**	Phase-transition material Calcium carbonate	US-triggered release Physical shock High-intensity focused ultrasound therapy
**Optical or** **optoacoustic**	NIR dyes	Photothermal therapy
	Metallic and inorganic NPs, e.g., gold nanorod, quantum dots	Photodynamic therapy

**Table 3:** Cancer nanotheranostics in clinical trials.

Drug Name	Composition	Imaging Label (Modality)	Therapeutic Agent (Mechanism)	NCT	Phase(s)	Cancer Type
**[^64^Cu]MM-302**	HER2-targeted ^64^Cu-labeled liposome containing doxorubicin	^64^Cu (PET)	Doxorubicin (chemotherapy)	NCT01304797	I	Breast cancer
				NCT02213744	II	
**[^89^Zr]-Df-CriPec^®^**	^89^Zr labeled micellar docetaxel conjugate	^89^Zr (PET)	Docetaxel (chemotherapy)	NCT03712423	I	Solid Tumor
**AGuIX^®^**	Polysiloxane matrix nanoparticles with Gd chelates	Gd (MRI)	Gd (radiosensitizer)	NCT02820454	I	Multiple brain metastases
				NCT03818386	II	
				NCT04899908	II	
				NCT03308604	I	Locally advanced cervical cancer
				NCT04881032	I/II	Newly Diagnosed Glioblastoma
**NBTXR3**	Hafnium oxide nanoparticles	Hafnium oxide (CT)	Hafnium oxide (radioenhancer)	NCT01433068	I	Soft tissue sarcoma
				NCT02379845	II/III	
				NCT02805894	I/II	Prostate adenocarcinoma
				NCT04505267	I	Non-small cell lung cancer
				NCT04834349	II	Head and neck squamous cell cancer (inoperable or recurrent)
				NCT04484909	I	Pancreatic cancer
				NCT04615013	I	Esophageal adenocarcinoma
				NCT04862455	II	Head and neck squamous cancer (recurrent or metastatic)
				NCT05039632	II	
				NCT04892173	III	Locally advanced squamous cell carcinoma

## Data Availability

Not applicable.

## References

[1] Thakor A.S., Gambhir S.S. (2013). Nanooncology: The future of cancer diagnosis and therapy. CA A Cancer J. Clin..

[2] Greene M.K., Johnston M.C., Scott C.J. (2021). Nanomedicine in Pancreatic Cancer: Current Status and Future Opportunities for Overcoming Therapy Resistance. Cancers.

[3] Ceci C., Lacal P.M., Graziani G. (2022). Antibody-drug conjugates: Resurgent anticancer agents with multi-targeted therapeutic potential. Pharmacol. Ther..

[4] Damiano M.G., Mutharasan R.K., Tripathy S., McMahon K.M., Thaxton C.S. (2013). Templated high density lipoprotein nanoparticles as potential therapies and for molecular delivery. Adv. Drug Deliv. Rev..

[5] Choi I.-K., Strauss R., Richter M., Yun C.-O., Lieber A. (2013). Strategies to Increase Drug Penetration in Solid Tumors. Front. Oncol..

[6] Yan H., Endo Y., Shen Y., Rotstein D., Dokmanovic M., Mohan N., Mukhopadhyay P., Gao B., Pacher P., Wu W.J. (2015). Ado-Trastuzumab Emtansine Targets Hepatocytes Via Human Epidermal Growth Factor Receptor 2 to Induce Hepatotoxicity. Mol. Cancer Ther..

[7] Dadwal A., Baldi A., Kumar Narang R. (2018). Nanoparticles as carriers for drug delivery in cancer. Artif. Cells Nanomed. Biotechnol..

[8] Frickenstein A., Hagood J., Britten C., Abbott B., McNally M., Vopat C., Patterson E., MacCuaig W., Jain A., Walters K. (2021). Mesoporous Silica Nanoparticles: Properties and Strategies for Enhancing Clinical Effect. Pharmaceutics.

[9] Zuazu-Jausoro I., Borrell M., Urrutia T., Oliver A., Montserrat I., Mateo J., Ribera L., Fontcuberta J. (1990). Detection of thrombin-antithrombin complexes in hypercoagulability conditions. Analysis of 182 cases. Sangre.

[10] Hostetler M.J., Wingate J.E., Zhong C.-J., Harris J.E., Vachet R.W., Clark M.R., Londono J.D., Green S.J., Stokes J.J., Wignall G.D. (1998). Alkanethiolate Gold Cluster Molecules with Core Diameters from 1.5 to 5.2 nm: Core and Monolayer Properties as a Function of Core Size. Langmuir.

[11] Shi J., Kantoff P.W., Wooster R., Farokhzad O.C. (2017). Cancer nanomedicine: Progress, challenges and opportunities. Nat. Rev. Cancer.

[12] Hare J.I., Lammers T., Ashford M.B., Puri S., Storm G., Barry S.T. (2017). Challenges and strategies in anti-cancer nanomedicine development: An industry perspective. Adv. Drug Deliv. Rev..

[13] Wilhelm S., Tavares A.J., Dai Q., Ohta S., Audet J., Dvorak H.F., Chan W.C.W. (2016). Analysis of nanoparticle delivery to tumours. Nat. Rev. Mater..

[14] Dai Q., Wilhelm S., Ding D., Syed A., Sindhwani S., Zhang Y., Chen Y.Y., MacMillan P., Chan W.C.W. (2018). Quantifying the Ligand-Coated Nanoparticle Delivery to Cancer Cells in Solid Tumors. ACS Nano.

[15] Dewhirst M.W., Secomb T.W. (2017). Transport of drugs from blood vessels to tumour tissue. Nat. Cancer.

[16] Manzoor A.A., Lindner L.H., Landon C.D., Park J.-Y., Simnick A.J., Dreher M.R., Das S., Hanna G., Park W., Chilkoti A. (2012). Overcoming Limitations in Nanoparticle Drug Delivery: Triggered, Intravascular Release to Improve Drug Penetration into Tumors. Cancer Res..

[17] Zhang J., Shin M.C., David A.E., Zhou J., Lee K., He H., Yang V.C. (2013). Long-Circulating Heparin-Functionalized Magnetic Nanoparticles for Potential Application as a Protein Drug Delivery Platform. Mol. Pharm..

[18] Li J., Liu F., Gupta S., Li C. (2016). Interventional Nanotheranostics of Pancreatic Ductal Adenocarcinoma. Theranostics.

[19] Cheng Z., Al Zaki A., Hui J.Z., Muzykantov V.R., Tsourkas A. (2012). Multifunctional Nanoparticles: Cost Versus Benefit of Adding Targeting and Imaging Capabilities. Science.

[20] Zhang W., Zang Y., Lu Y., Han J., Xiong Q., Xiong J. (2022). Photothermal Effect and Multi-Modality Imaging of Up-Conversion Nanomaterial Doped with Gold Nanoparticles. Int. J. Mol. Sci..

[21] Jiang K., Zhang L., Bao G. (2021). Magnetic Iron Oxide Nanoparticles for Biomedical Applications. Curr. Opin. Biomed. Eng..

[22] Anselmo A.C., Mitragotri S. (2019). Nanoparticles in the clinic: An update. Bioeng. Transl. Med..

[23] Mitchell M.J., Billingsley M.M., Haley R.M., Wechsler M.E., Peppas N.A., Langer R. (2020). Engineering precision nanoparticles for drug delivery. Nat. Rev. Drug Discov..

[24] Öztürk K., Esendagli G., Gürbüz M.U., Tülü M., Çalış S. (2017). Effective targeting of gemcitabine to pancreatic cancer through PEG-cored Flt-1 antibody-conjugated dendrimers. Int. J. Pharm..

[25] Mekuria S.L., Ouyang Z., Song C., Rodrigues J., Shen M., Shi X. (2021). Dendrimer-Based Nanogels for Cancer Nanomedicine Applications. Bioconjugate Chem..

[26] Liu X., Situ A., Kang Y., Villabroza K.R., Liao Y., Chang C.H., Donahue T., Nel A.E., Meng H. (2016). Irinotecan Delivery by Lipid-Coated Mesoporous Silica Nanoparticles Shows Improved Efficacy and Safety over Liposomes for Pancreatic Cancer. ACS Nano.

[27] Yang F., Jin C., Yang D., Jiang Y., Li J., Di Y., Hu J., Wang C., Ni Q., Fu D. (2011). Magnetic functionalised carbon nanotubes as drug vehicles for cancer lymph node metastasis treatment. Eur. J. Cancer.

[28] Rosenberger I., Strauss A., Dobiasch S., Weis C., Szanyi S., Gil-Iceta L., Alonso E., Esparza M.G., Vallejo V.G., Szczupak B. (2015). Targeted diagnostic magnetic nanoparticles for medical imaging of pancreatic cancer. J. Control Release.

[29] Nigam P., Waghmode S., Louis M., Wangnoo S., Chavan P., Sarkar D. (2014). Graphene quantum dots conjugated albumin nanoparticles for targeted drug delivery and imaging of pancreatic cancer. J. Mater. Chem. B.

[30] Balfourier A., Kolosnjaj-Tabi J., Luciani N., Carn F., Gazeau F. (2020). Gold-based therapy: From past to present. Proc. Natl. Acad. Sci. USA.

[31] Libutti S.K., Paciotti G.F., Byrnes A.A., Alexander H.R., Gannon W.E., Walker M., Seidel G.D., Yuldasheva N., Tamarkin L. (2010). Phase I and pharmacokinetic studies of CYT-6091, a novel PEGylated colloidal gold-rhTNF nanomedicine. Clin. Cancer Res..

[32] Ray P., Confeld M., Borowicz P., Wang T., Mallik S., Quadir M. (2019). PEG-b-poly(carbonate)-derived nanocarrier platform with pH-responsive properties for pancreatic cancer combination therapy. Colloids Surf. B Biointerfaces.

[33] Lee G.Y., Qian W.P., Wang L., Wang Y.A., Staley C.A., Satpathy M., Nie S., Mao H., Yang L. (2013). Theranostic Nanoparticles with Controlled Release of Gemcitabine for Targeted Therapy and MRI of Pancreatic Cancer. ACS Nano.

[34] Dadfar S.M., Roemhild K., Drude N., von Stillfried S., Knüchel R., Kiessling F., Lammers T. (2019). Iron oxide nanoparticles: Diagnostic, therapeutic and theranostic applications. Adv. Drug Deliv. Rev..

[35] Mattheolabakis G., Milane L., Singh A.P., Amiji M.M. (2015). Hyaluronic acid targeting of CD44 for cancer therapy: From receptor biology to nanomedicine. J. Drug Target..

[36] Tummers W.S., Miller S.E., Teraphongphom N.T., Gomez A., Steinberg I., Huland D.M., Hong S., Kothapalli S.-R., Hasan A., Ertsey R. (2018). Intraoperative Pancreatic Cancer Detection using Tumor-Specific Multimodality Molecular Imaging. Ann. Surg. Oncol..

[37] Wang M., Li Y., Wang M., Liu K., Hoover A.R., Li M., Towner R.A., Mukherjee P., Zhou F., Qu J. (2022). Synergistic interventional photothermal therapy and immunotherapy using an iron oxide nanoplatform for the treatment of pancreatic cancer. Acta Biomater.

[38] Alavi M., Karimi N., Safaei M. (2017). Application of Various Types of Liposomes in Drug Delivery Systems. Adv. Pharm. Bull..

[39] Xing H., Hwang K., Lu Y. (2016). Recent Developments of Liposomes as Nanocarriers for Theranostic Applications. Theranostics.

[40] Sercombe L., Veerati T., Moheimani F., Wu S.Y., Sood A.K., Hua S. (2015). Advances and Challenges of Liposome Assisted Drug Delivery. Front. Pharmacol..

[41] Sarfraz M., Afzal A., Yang T., Gai Y., Raza S.M., Khan M.W., Cheng Y., Ma X., Xiang G. (2018). Development of Dual Drug Loaded Nanosized Liposomal Formulation by A Reengineered Ethanolic Injection Method and Its Pre-Clinical Pharmacokinetic Studies. Pharmaceutics.

[42] Phillips W.T., Bao A., Sou K., Li S., Goins B. (2013). Radiolabeled liposomes as drug delivery nanotheranostics. Drug Delivery Applications of Noninvasive Imaging Validation from Biodistribution to Sites of Action.

[43] Petersen A.L., Hansen A.E., Gabizon A., Andresen T.L. (2012). Liposome imaging agents in personalized medicine. Adv. Drug Deliv. Rev..

[44] Zhang K., Zhang Y., Meng X., Lu H., Chang H., Dong H., Zhang X. (2018). Light-triggered theranostic liposomes for tumor diagnosis and combined photodynamic and hypoxia-activated prodrug therapy. Biomaterials.

[45] Karpuz M., Silindir-Gunay M., Ozer A.Y., Ozturk S.C., Yanik H., Tuncel M., Aydin C., Esendagli G. (2020). Diagnostic and therapeutic evaluation of folate-targeted paclitaxel and vinorelbine encapsulating theranostic liposomes for non-small cell lung cancer. Eur. J. Pharm. Sci..

[46] Maeda H. (2012). Macromolecular therapeutics in cancer treatment: The EPR effect and beyond. J. Control. Release.

[47] Handali S., Moghimipour E., Kouchak M., Ramezani Z., Amini M., Angali K.A., Saremy S., Dorkoosh F.A., Rezaei M. (2019). New folate receptor targeted nano liposomes for delivery of 5-fluorouracil to cancer cells: Strong implication for enhanced potency and safety. Life Sci..

[48] Thomas A., Samykutty A., Gomez-Gutierrez J.G., Yin W., Egger M.E., McNally M., Chuong P., MacCUAIG W.M., Albeituni S., Zeiderman M. (2020). Actively Targeted Nanodelivery of Echinomycin Induces Autophagy-Mediated Death in Chemoresistant Pancreatic Cancer In Vivo. Cancers.

[49] Aghebati-Maleki A., Dolati S., Ahmadi M., Baghbanzhadeh A., Asadi M., Fotouhi A., Yousefi M., Aghebati-Maleki L. (2020). Nanoparticles and cancer therapy: Perspectives for application of nanoparticles in the treatment of cancers. J. Cell Physiol..

[50] Siafaka P.I., Okur N., Karavas E., Bikiaris D.N. (2016). Surface Modified Multifunctional and Stimuli Responsive Nanoparticles for Drug Targeting: Current Status and Uses. Int. J. Mol. Sci..

[51] Rideau E., Dimova R., Schwille P., Wurm F.R., Landfester K. (2018). Liposomes and polymersomes: A comparative review towards cell mimicking. Chem. Soc. Rev..

[52] Zhang Y., He P., Zhang P., Yi X., Xiao C., Chen X. (2021). Polypeptides–Drug Conjugates for Anticancer Therapy. Adv. Health Mater..

[53] Gustafson H.H., Holt-Casper D., Grainger D.W., Ghandehari H. (2015). Nanoparticle uptake: The phagocyte problem. Nano Today.

[54] Varani M., Campagna G., Bentivoglio V., Serafinelli M., Martini M.L., Galli F., Signore A. (2021). Synthesis and Biodistribution of ^99m^Tc-Labeled PLGA Nanoparticles by Microfluidic Technique. Pharmaceutics.

[55] Huang G., Zhao T., Wang C., Nham K., Xiong Y., Gao X., Wang Y., Hao G., Ge W.P., Sun X. (2020). PET imaging of occult tumours by temporal integration of tumour-acidosis signals from pH-sensitive (64)Cu-labelled polymers. Nat. Biomed. Eng..

[56] Carvalho M.R., Reis R.L., Oliveira J.M. (2020). Dendrimer nanoparticles for colorectal cancer applications. J. Mater. Chem. B.

[57] Nottelet B., Darcos V., Coudane J. (2015). Aliphatic polyesters for medical imaging and theranostic applications. Eur. J. Pharm. Biopharm..

[58] Aso E., Martinsson I., Appelhans D., Effenberg C., Benseny-Cases N., Cladera J., Gouras G., Ferrer I., Klementieva O. (2019). Poly(propylene imine) dendrimers with histidine-maltose shell as novel type of nanoparticles for synapse and memory protection. Nanomed. Nanotechnol. Biol. Med..

[59] Singh V., Kesharwani P. (2021). Dendrimer as a promising nanocarrier for the delivery of doxorubicin as an anticancer therapeutics. J. Biomater. Sci. Polym. Ed..

[60] Lesniak W.G., Boinapally S., Banerjee S.R., Azad B.B., Foss C.A., Shen C., Lisok A., Wharram B., Nimmagadda S., Pomper M.G. (2019). Evaluation of PSMA-Targeted PAMAM Dendrimer Nanoparticles in a Murine Model of Prostate Cancer. Mol. Pharm..

[61] Liu J., Liu J., Chu L., Wang Y., Duan Y., Feng L., Yang C., Wang L., Kong D. (2011). Novel peptide–dendrimer conjugates as drug carriers for targeting nonsmall cell lung cancer. Int. J. Nanomed..

[62] Marcinkowska M., Stanczyk M., Janaszewska A., Sobierajska E., Chworos A., Klajnert-Maculewicz B. (2019). Multicomponent Conjugates of Anticancer Drugs and Monoclonal Antibody with PAMAM Dendrimers to Increase Efficacy of HER-2 Positive Breast Cancer Therapy. Pharm. Res..

[63] Dias A.P., da Silva Santos S., da Silva J.V., Parise-Filho R., Ferreira E.I., El Seoud O., Giarolla J. (2020). Dendrimers in the context of nanomedicine. Int. J. Pharm..

[64] Toljic D., Angelovski G. (2019). Translating a Low-Molecular-Weight MRI Probe Sensitive to Amino Acid Neurotransmitters into a PAMAM Dendrimer Conjugate: The Impact of Conjugation. ChemNanoMat.

[65] Almasi T., Gholipour N., Akhlaghi M., Kheirabadi A.M., Mazidi S.M., Hosseini S.H., Geramifar P., Beiki D., Rostampour N., Gahrouei D.S. (2020). Development of Ga-68 radiolabeled DOTA functionalized and acetylated PAMAM dendrimer-coated iron oxide nanoparticles as PET/MR dual-modal imaging agent. Int. J. Polym. Mater. Polym. Biomater..

[66] Sun N., Zhao L., Zhu J., Li Y., Song N., Xing Y., Qiao W., Huang H., Zhao J. (2019). 131I-labeled polyethylenimine-entrapped gold nanoparticles for targeted tumor SPECT/CT imaging and radionuclide therapy. Int. J. Nanomed..

[67] Shreffler J.W., Pullan J.E., Dailey K.M., Mallik S., Brooks A.E. (2019). Overcoming Hurdles in Nanoparticle Clinical Translation: The Influence of Experimental Design and Surface Modification. Int. J. Mol. Sci..

[68] Dubey S.K., Bhatt T., Agrawal M., Saha R.N., Saraf S., Saraf S., Alexander A. (2022). Application of chitosan modified nanocarriers in breast cancer. Int. J. Biol. Macromol..

[69] Sampath M., Pichaimani A., Kumpati P., Sengottuvelan B. (2020). The remarkable role of emulsifier and chitosan, dextran and PEG as capping agents in the enhanced delivery of curcumin by nanoparticles in breast cancer cells. Int. J. Biol. Macromol..

[70] Wang F.H., Bae K., Huang Z.W., Xue J.M. (2018). Two-photon graphene quantum dot modified Gd_2_O_3_ nanocomposites as a dual-mode MRI contrast agent and cell labelling agent. Nanoscale.

[71] Tanaka S., Lin J., Kaneti Y.V., Yusa S.-I., Jikihara Y., Nakayama T., Zakaria M.B., Alshehri A.A., You J., Hossain S.A. (2018). Gold nanoparticles supported on mesoporous iron oxide for enhanced CO oxidation reaction. Nanoscale.

[72] Motiei M., Dreifuss T., Sadan T., Omer N., Blumenfeld-Katzir T., Fragogeorgi E., Loudos G., Popovtzer R., Ben-Eliezer N. (2019). Trimodal nanoparticle contrast agent for ct, mri and spect imaging: Synthesis and characterization of radiolabeled core/shell iron oxide@ gold nanoparticles. Chem. Lett..

[73] Atukorale P.U., Covarrubias G., Bauer L., Karathanasis E. (2016). Vascular targeting of nanoparticles for molecular imaging of diseased endothelium. Adv. Drug Deliv. Rev..

[74] Molema G. (2002). Tumor vasculature directed drug targeting: Applying new technologies and knowledge to the development of clinically relevant therapies. Pharm. Res..

[75] Stylianopoulos T., Martin J., Snuderl M., Mpekris F., Jain S.R., Jain R.K. (2013). Coevolution of Solid Stress and Interstitial Fluid Pressure in Tumors during Progression: Implications for Vascular Collapse. Cancer Res..

[76] Fernandes C., Suares D., Yergeri M.C. (2018). Tumor Microenvironment Targeted Nanotherapy. Front. Pharmacol..

[77] MacCuaig W.M., Fouts B.L., McNally M.W., Grizzle W.E., Chuong P., Samykutty A., Mukherjee P., Li M., Jasinski J.B., Behkam B. (2021). Active Targeting Significantly Outperforms Nanoparticle Size in Facilitating Tumor-Specific Uptake in Orthotopic Pancreatic Cancer. ACS Appl. Mater. Interfaces.

[78] Serri C., Quagliariello V., Iaffaioli R.V., Fusco S., Botti G., Mayol L., Biondi M. (2018). Combination therapy for the treatment of pancreatic cancer through hyaluronic acid-decorated nanoparticles loaded with quercetin and gemcitabine: A preliminary in vitro study. J. Cell. Physiol..

[79] Herting C.J., Karpovsky I., Lesinski G.B. (2021). The tumor microenvironment in pancreatic ductal adenocarcinoma: Current perspectives and future directions. Cancer Metastasis Rev..

[80] Zhang Y.-F., Jiang S.-H., Hu L.-P., Huang P.-Q., Wang X., Li J., Zhang X.-L., Nie H.-Z., Zhang Z.-G. (2019). Targeting the tumor microenvironment for pancreatic ductal adenocarcinoma therapy. Chin. Clin. Oncol..

[81] Man F., Lammers T., De Rosales R.T.M. (2018). Imaging Nanomedicine-Based Drug Delivery: A Review of Clinical Studies. Mol. Imaging Biol..

[82] Cuaron J., Hirsch J., Medich D., Rosenstein B., Martel C., Hirsch A. (2009). A Proposed Methodology to Select Radioisotopes for Use in Radionuclide Therapy. Am. J. Neuroradiol..

[83] Lipowska M., Klenc J., Taylor A.T., Marzilli L.G. (2018). fac-99mTc/Re-tricarbonyl complexes with tridentate aminocarboxyphosphonate ligands: Suitability of the phosphonate group in chelate ligand design of new imaging agents. Inorg. Chim. Acta.

[84] Wadas T.J., Wong E.H., Weisman G.R., Anderson C.J. (2010). Coordinating Radiometals of Copper, Gallium, Indium, Yttrium, and Zirconium for PET and SPECT Imaging of Disease. Chem. Rev..

[85] Ni D., Jiang D., Ehlerding E.B., Huang P., Cai W. (2018). Radiolabeling Silica-Based Nanoparticles via Coordination Chemistry: Basic Principles, Strategies, and Applications. Acc. Chem. Res..

[86] Good S., Walter M.A., Waser B., Wang X., Müller-Brand J., Béhé M.P., Reubi J.-C., Maecke H.R. (2008). Macrocyclic chelator-coupled gastrin-based radiopharmaceuticals for targeting of gastrin receptor-expressing tumours. Eur. J. Pediatr..

[87] Chang A.J., Sohn R., Lu Z.H., Arbeit J.M., Lapi S.E. (2013). Detection of Rapalog-Mediated Therapeutic Response in Renal Cancer Xenografts Using ^64^Cu-bevacizumab ImmunoPET. PLoS ONE.

[88] Shokeen M., Anderson C.J. (2009). Molecular Imaging of Cancer with Copper-64 Radiopharmaceuticals and Positron Emission Tomography (PET). Acc. Chem. Res..

[89] Zhang Y., Hong H., Engle J.W., Bean J., Yang Y., Leigh B.R., Barnhart T.E., Cai W. (2011). Positron Emission Tomography Imaging of CD105 Expression with a ^64^Cu-Labeled Monoclonal Antibody: NOTA Is Superior to DOTA. PLoS ONE.

[90] Rauscher A., Frindel M., Rajerison H., Gouard S., Maurel C., Barbet J., Faivre-Chauvet A., Mougin-Degraef M. (2015). Improvement of the Targeting of Radiolabeled and Functionalized Liposomes with a Two-Step System Using a Bispecific Monoclonal Antibody (Anti-CEA x Anti-DTPA-In). Front. Med..

[91] Borràs J., Mesa V., Suades J., Barnadas-Rodriguez R. (2020). Direct Synthesis of Rhenium and Technetium-99m Metallosurfactants by a Transmetallation Reaction of Lipophilic Groups: Potential Applications in the Radiolabeling of Liposomes. Langmuir.

[92] Aranda-Lara L., Morales-Avila E., Luna-Gutiérrez M.A., Olivé-Alvarez E., Isaac-Olivé K. (2020). Radiolabeled liposomes and lipoproteins as lipidic nanoparticles for imaging and therapy. Chem. Phys. Lipids.

[93] Petersen A.L., Binderup T., Rasmussen P., Henriksen J.R., Elema D.R., Kjaer A., Andresen T.L. (2011). ^64^Cu loaded liposomes as positron emission tomography imaging agents. Biomaterials.

[94] Engudar G., Schaarup-Jensen H., Fliedner F.P., Hansen A.E., Kempen P., Jølck R.I., Kjaer A., Andresen T.L., Clausen M.H., Jensen A. (2018). Remote loading of liposomes with a 124I-radioiodinated compound and their in vivo evaluation by PET/CT in a murine tumor model. Theranostics.

[95] Lee H., Shields A.F., Siegel B.A., Miller K.D., Krop I., Ma C.X., LoRusso P.M., Munster P.N., Campbell K., Gaddy D.F. (2017). ^64^Cu-MM-302 Positron Emission Tomography Quantifies Variability of Enhanced Permeability and Retention of Nanoparticles in Relation to Treatment Response in Patients with Metastatic Breast Cancer. Clin. Cancer Res..

[96] Pratt E.C., Shaffer T.M., Grimm J. (2016). Nanoparticles and radiotracers: Advances toward radionanomedicine. Wiley Interdiscip. Rev. Nanomed. Nanobiotechnol..

[97] Shi S., Xu C., Yang K., Goel S., Valdovinos H., Luo H., Ehlerding E.B., England C.G., Cheng L., Chen F. (2017). Chelator-Free Radiolabeling of Nanographene: Breaking the Stereotype of Chelation. Angew. Chem. Int. Ed..

[98] Wall M.A., Shaffer T.M., Harmsen S., Tschaharganeh D.-F., Huang C.-H., Lowe S.W., Drain C.M., Kircher M.F. (2017). Chelator-Free Radiolabeling of SERRS Nanoparticles for Whole-Body PET and Intraoperative Raman Imaging. Theranostics.

[99] Tang T., Wei Y., Yang Q., Yang Y., Sailor M.J., Pang H.-B. (2019). Rapid chelator-free radiolabeling of quantum dots for in vivo imaging. Nanoscale.

[100] Miller K., Cortes J., Hurvitz S.A., Krop I.E., Tripathy D., Verma S., Riahi K., Reynolds J.G., Wickham T.J., Molnar I. (2016). HERMIONE: A randomized Phase 2 trial of MM-302 plus trastuzumab versus chemotherapy of physician’s choice plus trastuzumab in patients with previously treated, anthracycline-naive, HER2-positive, locally advanced/metastatic breast cancer. BMC Cancer.

[101] Lee H., Zheng J., Gaddy D., Orcutt K.D., Leonard S., Geretti E., Hesterman J., Harwell C., Hoppin J., Jaffray D.A. (2015). A gradient-loadable ^64^Cu-chelator for quantifying tumor deposition kinetics of nanoliposomal therapeutics by positron emission tomography. Nanomed. Nanotechnol. Biol. Med..

[102] Jentzen W., Verschure F., van Zon A., van de Kolk R., Wierts R., Schmitz J., Bockisch A., Binse I. (2016). 124I PET Assessment of Response of Bone Metastases to Initial Radioiodine Treatment of Differentiated Thyroid Cancer. J. Nucl. Med..

[103] Lopci E., Chiti A., Castellani M.R., Pepe G., Antunovic L., Fanti S., Bombardieri E. (2011). Matched pairs dosimetry: 124I/131I metaiodobenzylguanidine and 124I/131I and 86Y/90Y antibodies. Eur. J. Pediatr..

[104] Marsh I.R., Grudzinski J.J., Baiu D.C., Besemer A., Hernandez R., Jeffery J.J., Weichert J.P., Otto M., Bednarz B.P. (2019). Preclinical Pharmacokinetics and Dosimetry Studies of 124I/131I-CLR1404 for Treatment of Pediatric Solid Tumors in Murine Xenograft Models. J. Nucl. Med..

[105] Goel S., Ferreira C.A., Dogra P., Yu B., Kutyreff C.J., Siamof C.M., Engle J.W., Barnhart T.E., Cristini V., Wang Z. (2019). Size-Optimized Ultrasmall Porous Silica Nanoparticles Depict Vasculature-Based Differential Targeting in Triple Negative Breast Cancer. Small.

[106] Ferreira C.A., Goel S., Ehlerding E.B., Rosenkrans Z.T., Jiang D., Sun T., Aluicio-Sarduy E., Engle J.W., Ni D., Cai W. (2021). Ultrasmall Porous Silica Nanoparticles with Enhanced Pharmacokinetics for Cancer Theranostics. Nano Lett..

[107] Imlimthan S., Khng Y.C., Keinänen O., Zhang W., Airaksinen A.J., Kostiainen M.A., Zeglis B.M., Santos H.A., Sarparanta M. (2021). A Theranostic Cellulose Nanocrystal-Based Drug Delivery System with Enhanced Retention in Pulmonary Metastasis of Melanoma. Small.

[108] Gaikwad G., Rohra N., Kumar C., Jadhav S., Sarma H.D., Borade L., Chakraborty S., Bhagwat S., Dandekar P., Jain R. (2021). A facile strategy for synthesis of a broad palette of intrinsically radiolabeled chitosan nanoparticles for potential use in cancer theranostics. J. Drug Deliv. Sci. Technol..

[109] Tweedle M.F., Wedeking P., Kumar K. (1995). Biodistribution of Radiolabeled, Formulated Gadopentetate, Gadoteridol, Gadoterate, and Gadodiamide in Mice and Rats. Investig. Radiol..

[110] Verry C., Dufort S., Villa J., Gavard M., Iriart C., Grand S., Charles J., Chovelon B., Cracowski J.-L., Quesada J.-L. (2021). Theranostic AGuIX nanoparticles as radiosensitizer: A phase I, dose-escalation study in patients with multiple brain metastases (NANO-RAD trial). Radiother. Oncol..

[111] Lux F., Tran V.L., Thomas E., Dufort S., Rossetti F., Martini M., Truillet C., Doussineau T., Bort G., Denat F. (2019). AGuIX((R)) from bench to bedside-Transfer of an ultrasmall theranostic gadolinium-based nanoparticle to clinical medicine. Br. J. Radiol..

[112] Sancey L., Lux F., Kotb S., Roux S., Dufort S., Bianchi A., Crémillieux Y., Fries P., Coll J.-L., Rodriguez-Lafrasse C. (2014). The use of theranostic gadolinium-based nanoprobes to improve radiotherapy efficacy. Br. J. Radiol..

[113] Verry C., Dufort S., Lemasson B., Grand S., Pietras J., Troprès I., Crémillieux Y., Lux F., Mériaux S., Larrat B. (2020). Targeting brain metastases with ultrasmall theranostic nanoparticles, a first-in-human trial from an MRI perspective. Sci. Adv..

[114] Gries M., Thomas N., Daouk J., Rocchi P., Choulier L., Jubréaux J., Pierson J., Reinhard A., Jouan-Hureaux V., Chateau A. (2020). Multiscale Selectivity and in vivo Biodistribution of NRP-1-Targeted Theranostic AGuIX Nanoparticles for PDT of Glioblastoma. Int. J. Nanomed..

[115] Chen H., Qiu Y., Ding D., Lin H., Sun W., Wang G.D., Huang W., Zhang W., Lee D., Liu G. (2018). Gadolinium-Encapsulated Graphene Carbon Nanotheranostics for Imaging-Guided Photodynamic Therapy. Adv. Mater..

[116] Guan M., Zhou Y., Liu S., Chen D., Ge J., Deng R., Li X., Yu T., Xu H., Sun D. (2019). Photo-triggered gadofullerene: Enhanced cancer therapy by combining tumor vascular disruption and stimulation of anti-tumor immune responses. Biomaterials.

[117] Lu Z., Jia W., Deng R., Zhou Y., Li X., Yu T., Zhen M., Wang C. (2020). Light-assisted gadofullerene nanoparticles disrupt tumor vasculatures for potent melanoma treatment. J. Mater. Chem. B.

[118] Han Z., Wu X., Roelle S., Chen C., Schiemann W.P., Lu Z.-R. (2017). Targeted gadofullerene for sensitive magnetic resonance imaging and risk-stratification of breast cancer. Nat. Commun..

[119] Si Y., Zhang G., Wang D., Zhang C., Yang C., Bai G., Qian J., Chen Q., Zhang Z., Wu Z. (2019). Nanostructure-enhanced water interaction to increase the dual-mode MR contrast performance of gadolinium-doped iron oxide nanoclusters. Chem. Eng. J..

[120] Guardia P., Di Corato R., Lartigue L., Wilhelm C., Espinosa A., Garcia-Hernandez M., Gazeau F., Manna L., Pellegrino T. (2012). Water-Soluble Iron Oxide Nanocubes with High Values of Specific Absorption Rate for Cancer Cell Hyperthermia Treatment. ACS Nano.

[121] Lartigue L., Innocenti C., Kalaivani T., Awwad A., Sanchez Duque M.D.M., Guari Y., Larionova J., Guérin C., Montero J.-L.G., Barragan-Montero V. (2011). Water-Dispersible Sugar-Coated Iron Oxide Nanoparticles. An Evaluation of their Relaxometric and Magnetic Hyperthermia Properties. J. Am. Chem. Soc..

[122] Hayashi K., Nakamura M., Sakamoto W., Yogo T., Miki H., Ozaki S., Abe M., Matsumoto T., Ishimura K. (2013). Superparamagnetic Nanoparticle Clusters for Cancer Theranostics Combining Magnetic Resonance Imaging and Hyperthermia Treatment. Theranostics.

[123] Al Faraj A., Shaik A.S., Al Sayed B. (2015). Preferential magnetic targeting of carbon nanotubes to cancer sites: Noninvasive tracking using MRI in a murine breast cancer model. Nanomedicine.

[124] Svenskaya Y., Garello F., Lengert E., Kozlova A., Verkhovskii R., Bitonto V., Ruggiero M.R., German S., Gorin D., Terreno E. (2021). Biodegradable polyelectrolyte/magnetite capsules for MR imaging and magnetic targeting of tumors. Nanotheranostics.

[125] Schleich N., Po C., Jacobs D., Ucakar B., Gallez B., Danhier F., Préat V. (2014). Comparison of active, passive and magnetic targeting to tumors of multifunctional paclitaxel/SPIO-loaded nanoparticles for tumor imaging and therapy. J. Control. Release.

[126] Yuan Y., Zhang J., Qi X., Li S., Liu G., Siddhanta S., Barman I., Song X., McMahon M.T., Bulte J.W.M. (2019). Furin-mediated intracellular self-assembly of olsalazine nanoparticles for enhanced magnetic resonance imaging and tumour therapy. Nat. Mater..

[127] Li Y., Chen H., Xu J., Yadav N.N., Chan K.W., Luo L., McMahon M., Vogelstein B., Van Zijl P.C., Zhou S. (2016). CEST theranostics: Label-free MR imaging of anticancer drugs. Oncotarget.

[128] Han Z., Li Y., Zhang J., Liu J., Chen C., Van Zijl P.C.M., Liu G. (2019). Molecular imaging of deoxycytidine kinase activity using deoxycytidine-enhanced CEST MRI. Cancer Res..

[129] Ngen E.J., Bar-Shir A., Jablonska A., Liu G., Song X., Ansari R., Bulte J.W.M., Janowski M., Pearl M., Walczak P. (2016). Imaging the DNA Alkylator Melphalan by CEST MRI: An Advanced Approach to Theranostics. Mol. Pharm..

[130] Han Z., Liu G. (2021). CEST MRI trackable nanoparticle drug delivery systems. Biomed. Mater..

[131] Castelli D.D., Terreno E., Longo D., Aime S. (2013). Nanoparticle-based chemical exchange saturation transfer (CEST) agents. NMR Biomed..

[132] Zhou L.-Q., Li P., Cui X.-W., Dietrich C.F. (2019). Ultrasound nanotheranostics in fighting cancer: Advances and prospects. Cancer Lett..

[133] Vallet-Regi M., Manzano M., Baeza A. (2018). Controlled Release with Emphasis on Ultrasound-Induced Release. Enzymes.

[134] Qin H., Teng R., Liu Y., Li J., Yu M. (2022). Drug Release from Gelsolin-Targeted Phase-Transition Nanoparticles Triggered by Low-Intensity Focused Ultrasound. Int. J. Nanomed..

[135] Novoselova M.V., German S.V., Abakumova T.O., Perevoschikov S.V., Sergeeva O.V., Nesterchuk M.V., Efimova O.I., Petrov K.S., Chernyshev V.S., Zatsepin T.S. (2021). Multifunctional nanostructured drug delivery carriers for cancer therapy: Multimodal imaging and ultrasound-induced drug release. Colloids Surf. B Biointerfaces.

[136] Yildirim A., Shi D., Roy S., Blum N.T., Chattaraj R., Cha J.N., Goodwin A.P. (2018). Nanoparticle-Mediated Acoustic Cavitation Enables High Intensity Focused Ultrasound Ablation Without Tissue Heating. ACS Appl. Mater. Interfaces.

[137] Sjöstrand S., Evertsson M., Jansson T. (2020). Magnetomotive Ultrasound Imaging Systems: Basic Principles and First Applications. Ultrasound Med. Biol..

[138] Qin S., Caskey C.F., Ferrara K.W. (2009). Ultrasound contrast microbubbles in imaging and therapy: Physical principles and engineering. Phys. Med. Biol..

[139] Bawiec C.R., Rosnitskiy P.B., Peek A.T., Maxwell A.D., Kreider W., ter Haar G.R., Sapozhnikov O.A., Khokhlova V.A., Khokhlova T.D. (2021). Inertial Cavitation Behaviors Induced by Nonlinear Focused Ultrasound Pulses. IEEE Trans. Ultrason. Ferroelectr. Freq. Control.

[140] Chowdhury S.M., Abou-Elkacem L., Lee T., Dahl J., Lutz A.M. (2020). Ultrasound and microbubble mediated therapeutic delivery: Underlying mechanisms and future outlook. J. Control Release.

[141] Hyvelin J.-M., Gaud E., Costa M., Helbert A., Bussat P., Bettinger T., Frinking P. (2017). Characteristics and Echogenicity of Clinical Ultrasound Contrast Agents: An In Vitro and In Vivo Comparison Study. J. Ultrasound Med..

[142] Wang X., Chen H., Zheng Y., Ma M., Chen Y., Zhang K., Zeng D., Shi J. (2012). Au-nanoparticle coated mesoporous silica nanocapsule-based multifunctional platform for ultrasound mediated imaging, cytoclasis and tumor ablation. Biomaterials.

[143] Li J., Ji H., Jing Y., Wang S. (2020). pH- and acoustic-responsive platforms based on perfluoropentane-loaded protein nanoparticles for ovarian tumor-targeted ultrasound imaging and therapy. Nanoscale Res. Lett..

[144] Lee J., Min H.-S., Gil You D., Kim K., Kwon I.C., Rhim T., Lee K.Y. (2016). Theranostic gas-generating nanoparticles for targeted ultrasound imaging and treatment of neuroblastoma. J. Control Release.

[145] Zhang X., Machuki J.O., Pan W., Cai W., Xi Z., Shen F., Zhang L., Yang Y., Gao F., Guan M. (2020). Carbon Nitride Hollow Theranostic Nanoregulators Executing Laser-Activatable Water Splitting for Enhanced Ultrasound/Fluorescence Imaging and Cooperative Phototherapy. ACS Nano.

[146] Pan X., Wang W., Huang Z., Liu S., Guo J., Zhang F., Yuan H., Li X., Liu F., Liu H. (2020). MOF-Derived Double-Layer Hollow Nanoparticles with Oxygen Generation Ability for Multimodal Imaging-Guided Sonodynamic Therapy. Angew. Chem. Int. Ed..

[147] Gao S., Wang G., Qin Z., Wang X., Zhao G., Ma Q., Zhu L. (2017). Oxygen-generating hybrid nanoparticles to enhance fluorescent/photoacoustic/ultrasound imaging guided tumor photodynamic therapy. Biomaterials.

[148] Wang P., Tang Q., Zhang L., Xu M., Sun L., Sun S., Zhang J., Wang S., Liang X. (2021). Ultrasmall Barium Titanate Nanoparticles for Highly Efficient Hypoxic Tumor Therapy via Ultrasound Triggered Piezocatalysis and Water Splitting. ACS Nano.

[149] McNally L.R., Mezera M., Morgan D.E., Frederick P.J., Yang E.S., Eltoum I.E., Grizzle W.E. (2016). Current and emerging clinical applications of multispectral optoacoustic tomography (MSOT) in oncology. Clin. Cancer Res..

[150] MacCuaig W.M., Jones M.A., Abeyakoon O., McNally L.R. (2020). Development of Multispectral Optoacoustic Tomography as a Clinically Translatable Modality for Cancer Imaging. Radiol. Imaging Cancer.

[151] Mantri Y., Jokerst J.V. (2020). Engineering Plasmonic Nanoparticles for Enhanced Photoacoustic Imaging. ACS Nano.

[152] Ilina K., MacCuaig W.M., Laramie M., Jeouty J.N., McNally L.R., Henary M. (2020). Squaraine Dyes: Molecular Design for Different Applications and Remaining Challenges. Bioconjugate Chem..

[153] Laramie M.D., Fouts B.L., MacCuaig W.M., Buabeng E., Jones M.A., Mukherjee P., Behkam B., McNally L.R., Henary M. (2021). Improved pentamethine cyanine nanosensors for optoacoustic imaging of pancreatic cancer. Sci. Rep..

[154] Samykutty A., Grizzle W.E., Fouts B.L., McNally M.W., Chuong P., Thomas A., Chiba A., Otali D., Woloszynska A., Said N. (2018). Optoacoustic imaging identifies ovarian cancer using a microenvironment targeted theranostic wormhole mesoporous silica nanoparticle. Biomaterials.

[155] Thomas A., Chiba A., Samykutty A., McNally M.W., McNally L.R. (2020). Tumor specific cargo release in ex vivo patient samples and murine models of triple negative breast cancer by a pH-targeted nanoparticle: V3-RUBY. Cancer Res..

[156] Khanal A., Ullum C., Kimbrough C.W., Garbett N.C., Burlison J.A., McNally M.W. (2015). Tumor targeted mesoporous silica-coated gold nanorods facilitate detection of pancreatic tumors using Multispectral optoacoustic tomography. Nano Res..

[157] Xie H., Liu M., You B., Luo G., Chen Y., Liu B., Jiang Z., Chu P.K., Shao J., Yu X.F. (2020). Biodegradable Bi_2_O_2_Se Quantum Dots for Photoacoustic Imaging-Guided Cancer Photothermal Therapy. Small.

[158] Wang Y., Gong N., Li Y., Lu Q., Wang X., Li J. (2019). Atomic-Level Nanorings (A-NRs) Therapeutic Agent for Photoacoustic Imaging and Photothermal/Photodynamic Therapy of Cancer. J. Am. Chem. Soc..

[159] Dai X., Zhao X., Liu Y., Chen B., Ding X., Zhao N., Xu F. (2021). Controlled Synthesis and Surface Engineering of Janus Chitosan-Gold Nanoparticles for Photoacoustic Imaging-Guided Synergistic Gene/Photothermal Therapy. Small.

[160] Wang D., Zhang Z., Lin L., Liu F., Wang Y., Guo Z., Li Y., Tian H., Chen X. (2019). Porphyrin-based covalent organic framework nanoparticles for photoacoustic imaging-guided photodynamic and photothermal combination cancer therapy. Biomaterials.

[161] Rostami A., Sazgarnia A. (2019). Gold nanoparticles as cancer theranostic agents. Nanomed. J..

[162] Curry T., Kopelman R., Shilo M., Popovtzer R. (2014). Multifunctional theranostic gold nanoparticles for targeted CT imaging and photothermal therapy. Contrast Media Mol. Imaging.

[163] Yang C., Guo C., Guo W., Zhao X., Liu S., Han X. (2018). Multifunctional Bismuth Nanoparticles as Theranostic Agent for PA/CT Imaging and NIR Laser-Driven Photothermal Therapy. ACS Appl. Nano Mater..

[164] Wei B., Zhang X., Zhang C., Jiang Y., Fu Y.-Y., Yu C., Sun S.-K., Yan X.-P. (2016). Facile Synthesis of Uniform-Sized Bismuth Nanoparticles for CT Visualization of Gastrointestinal Tract In Vivo. ACS Appl. Mater. Interfaces.

[165] Bagley A.F., Ludmir E.B., Maitra A., Minsky B.D., Smith G.L., Das P., Koong A.C., Holliday E.B., Taniguchi C.M., Katz M.H. (2022). NBTXR3, a first-in-class radioenhancer for pancreatic ductal adenocarcinoma: Report of first patient experience. Clin. Transl. Radiat. Oncol..

[166] Bonvalot S., Le Pechoux C., De Baere T., Kantor G., Buy X., Stoeckle E., Terrier P., Sargos P., Coindre J.M., Lassau N. (2016). First-in-Human Study Testing a New Radioenhancer Using Nanoparticles (NBTXR3) Activated by Radiation Therapy in Patients with Locally Advanced Soft Tissue Sarcomas. Clin. Cancer Res..

[167] Wu Y., Zhou I.Y., Igarashi T., Longo D., Aime S., Sun P.Z. (2017). A generalized ratiometric chemical exchange saturation transfer (CEST) MRI approach for mapping renal pH using iopamidol. Magn. Reson. Med..

[168] Chen Z., Li Y., Airan R., Han Z., Xu J., Chan K.W.Y., Xu Y., Bulte J.W.M., van Zijl P.C.M., McMahon M.T. (2019). CT and CEST MRI bimodal imaging of the intratumoral distribution of iodinated liposomes. Quant. Imaging Med. Surg..

[169] Anselmo A.C., Mitragotri S. (2021). Nanoparticles in the clinic: An update post COVID-19 vaccines. Bioeng. Transl. Med..

[170] Grueneisen J., Nagarajah J., Buchbender C., Hoffmann O., Schaarschmidt B.M., Poeppel T., Forsting M., Quick H.H., Umutlu L., Kinner S. (2015). Positron Emission Tomography/Magnetic Resonance Imaging for Local Tumor Staging in Patients with Primary Breast Cancer: A Comparison with Positron Emission Tomography/Computed Tomography and Magnetic Resonance Imaging. Invest. Radiol..

[171] Vannier M.W. (2009). CT clinical perspective: Challenges and the impact of future technology developments. Annu. Int. Conf. IEEE Eng. Med. Biol. Soc..

[172] Vandenberghe S., Marsden P.K. (2015). PET-MRI: A review of challenges and solutions in the development of integrated multimodality imaging. Phys. Med. Biol..

[173] Miao Y., Zhang H., Cai J., Chen Y., Ma H., Zhang S., Yi J.B., Liu X., Bay B.-H., Guo Y. (2021). Structure–Relaxivity Mechanism of an Ultrasmall Ferrite Nanoparticle T_1_ MR Contrast Agent: The Impact of Dopants Controlled Crystalline Core and Surface Disordered Shell. Nano Lett..

[174] Popov A.L., Abakumov M.A., Savintseva I.V., Ermakov A.M., Popova N.R., Ivanova O.S., Kolmanovich D.D., Baranchikov A.E., Ivanov V.K. (2021). Biocompatible dextran-coated gadolinium-doped cerium oxide nanoparticles as MRI contrast agents with high *T*_1_ relaxivity and selective cytotoxicity to cancer cells. J. Mater. Chem. B.

[175] Wu M., Shu J. (2018). Multimodal Molecular Imaging: Current Status and Future Directions. Contrast Media Mol. Imaging.

[176] Van Der Geest T., Laverman P., Gerrits D., Franssen G.M., Metselaar J.M., Storm G., Boerman O.C. (2015). Comparison of three remote radiolabelling methods for long-circulating liposomes. J. Control Release.

[177] Cho M.H., Shin S.H., Park S.H., Kadayakkara D.K., Kim D., Choi Y. (2019). Targeted, Stimuli-Responsive, and Theranostic ^19^F Magnetic Resonance Imaging Probes. Bioconjug. Chem..

[178] Zheng B., Yu E., Orendorff R., Lu K., Konkle J.J., Tay Z.W., Hensley D., Zhou X.Y., Chandrasekharan P., Saritas E.U. (2017). Seeing SPIOs Directly In Vivo with Magnetic Particle Imaging. Mol. Imaging Biol..

[179] Canetta E. (2021). Current and Future Advancements of Raman Spectroscopy Techniques in Cancer Nanomedicine. Int. J. Mol. Sci..

[180] Han Z., Liu S., Pei Y., Ding Z., Li Y., Wang X., Zhan D., Xia S., Driedonks T., Witwer K.W. (2021). Highly efficient magnetic labelling allows MRI tracking of the homing of stem cell-derived extracellular vesicles following systemic delivery. J. Extracell. Vesicles.

[181] Sun X., Hong Y., Gong Y., Zheng S., Xie D. (2021). Bioengineered Ferritin Nanocarriers for Cancer Therapy. Int. J. Mol. Sci..

[182] Wang H., Liu Y., He R., Xu D., Zang J., Weeranoppanant N., Dong H., Li Y. (2019). Cell membrane biomimetic nanoparticles for inflammation and cancer targeting in drug delivery. Biomater. Sci..

